# How Executive Functions Are Evaluated in Children and Adolescents with Cerebral Palsy? A Systematic Review

**DOI:** 10.3389/fpsyg.2018.00021

**Published:** 2018-02-06

**Authors:** Armanda Pereira, Sílvia Lopes, Paula Magalhães, Adriana Sampaio, Elisa Chaleta, Pedro Rosário

**Affiliations:** ^1^Department of Applied Psychology, CIPsi, School of Psychology, University of Minho, Braga, Portugal; ^2^Neuropsychophysiology Lab, CIPsi, School of Psychology, University of Minho, Braga, Portugal; ^3^Department of Psychology, CIEP, School of Social Science, University of Évora, Évora, Portugal

**Keywords:** cerebral palsy, executive functions, assessment, learning difficulties, systematic review

## Abstract

**Aims:** The aim of the present study was to examine how executive functions are assessed in children and adolescents with Cerebral Palsy.

**Method:** A systematic literature review was conducted using four bibliographic databases (WebScience, Scopus, PubMed, and Psycinfo), and only studies that evaluated at least one executive function were selected. Both the research and reporting of results were based on Cochrane's recommendations and PRISMA (Preferred Reporting Items for Systematic Reviews and Meta-Analysis) guidelines.

**Results:** The instrument most frequently used was the D-KEFS. All studies point to the existence of impairments in the executive functions among children and adolescents with Cerebral Palsy with an impact on several cognitive and life domains.

**Interpretation:** There is a need to further systematize the research protocols to study the executive functions and their assessment in the intervention context. Findings of this review presented a diversity of tests (e.g., D-KEFS) or tasks (e.g., The inhibitory ability task) used with children with Cerebral Palsy. However, no information was given about adaptations performed to the test/task to meet Cerebral Palsy's specificities. Future research could consider including this information, which is key both to researchers and practitioners. The results of this study have important implications and suggestions for future avenues and guidelines for research and practice.

## Introduction

Executive Functions (EFs) are conceptualized as a set of cognitive processes responsible for the individual to consciously self-regulate emotions and goal-directed actions (Luria, 1995; Zelazo and Müller, 2010; Diamond, 2013). Recent findings (Blair, 2013; Zelazo and Anderson, 2013; Zelazo et al., 2016) describe EFs as attention-regulation skills involved in processes such as self-regulation and cognitive control. In fact, EFs, as neurocognitive processes, are conceived as a regulatory mechanism of the mind (Miyake et al., 2000b; Zelazo and Anderson, 2013). Cognitive control processes allow individuals to command and modify their actions at their own discretion while considering the consequences of each behavior (Atkinson and Shiffrin, 1968; Zelazo and Anderson, 2013; Zelazo et al., 2016). EFs are related to the family of top-down processes involved in decision-making, anticipation of consequences, and sustained focus on a specific task (e.g., “I will not watch more TV because I must study for the next week's exam”). These top-down processes are interdependent with bottom-up processes, which are more automatic and more responsive to emotional and physiological stimulation (e.g., “To me, exams cause great anxiety”) (Diamond, 2013; Zelazo et al., 2016). In sum, EFs are likely to have a significant impact on the development of everyday life skills; for example, EFs improve school achievement and vice-versa.

Over the years, several tentative models that integrate EFs have been introduced. Luria (1995) was the first to conceptualize and define EFs, but only recently have more complete models arisen. In fact, empirically-based models have gained attention in the literature (e.g., Miyake et al., 2000b; Anderson, 2002; Diamond, 2013), with the model conceptualization following different theoretical lines. For example, in Miyake et al. (2000b) factor-analytic work, EFs are conceived as a collection of three partially independent latent variables. These latent variables, “unity and diversity of EFs” (p. 87), represent the different roles that EFs have in complex cognition processes which can be captured through the measures of EF (Inhibition, Shifting, and Updating) in an individual's performance (Miyake et al., 2000a). Conversely, Anderson (2002) postulated a four-structure model of EFs, including the functions of Attentional Control, Cognitive Flexibility, Goal Setting, and Information Processing, which was conceived as being interrelated and integrative as a whole. Recently, Cunningham and Zelazo (2007) presented the Iterative Reprocessing model, which postulates that the goal-directed control of attention is verbally mediated by working memory through formulation and maintenance of rules.

Literature addressing EFs faces challenges regarding the difficulty of introducing precise definitions of the construct itself (Miyake et al., 2000b; Zelazo et al., 2016). EFs have been conceived as a multidimensional theoretical construct composed by different cognitive processes. Moreover, these cognitive processes are not easily assessed because they are diverse and they occupy different evolutionary stages and timeframes of human development (Gnys and Willis, 1991; Miyake et al., 2000b; Anderson, 2002; Hughes and Graham, 2002; Smidts et al., 2003; Romine and Reynolds, 2005). Besides, literature faces another conceptual challenge concerning the difference between executive function and executive functioning. The term “executive function” is likely to conceptualize each EF as interrelated, but each component is considered independent (e.g., Miyake et al., 2000a). Contrastingly, the term “executive functioning” is often conceptualized as an interrelated and interdependent multi-process related system (e.g., Anderson, 2002). Specifically, the executive function may be assessed by tasks or measures focusing on a specific function (e.g., inhibition), and the result allows for the identification of the functions that are in need of training or stimulation. Conversely, the executive functioning may be assessed through the relationship between these functions and their expression (e.g., everyday behaviors; Bull and Scerif, 2001; Isquith et al., 2004).

The scientific community has not yet agreed on the tasks best suited to measure the EFs (Miyake et al., 2000b). Furthermore, the tasks that measure EFs performance usually assess, simultaneously, the outcomes of several distinct and partially overlapping operating processes (Zelazo et al., 2016). This “task impurity” (Miyake et al., 2000b, p. 174) feature of the assessment process is of great concern among researchers in the EFs domain. The standardization of evaluation protocols and tasks used to measure EFs is expected to minimize the effect of this “task impurity”, as well as of other obstacles associated with the assessment of EFs (e.g., low-reliability problem; Miyake et al., 2000b; Hughes and Graham, 2002). For example, the use of multiple measures to contrast the outcomes of each executive function (EF) is referred to as a good practice (Miyake et al., 2000b). However, as Miyake et al. (2000b) stated, there is always an “impurity” linked to each task (p. 174).

Cerebral Palsy (CP) is the most common physical childhood disorder (Novak et al., 2013); therefore, it is relevant to further extend the knowledge on the primary (e.g., motor) and secondary (e.g., learning disabilities) impairments related with this clinical condition.

CP is a neurological, non-progressive, and permanent developmental disorder that mainly affects movement and posture (Bax et al., 2005; Rosenbaum et al., 2007). Its prevalence, 1.5–2.5 children per 1000 live births (Surveillance of Cerebral Palsy in Europe, 2002), is slightly increasing due to the higher number of premature infants' survival (Paneth et al., 2006). CP's motor impairments are often accompanied by disturbances in sensation, perception, cognition, communication, and behavior, as well as the presence of epilepsy, disequilibrium (Oskoui et al., 2013), and secondary musculoskeletal problems (Rosenbaum et al., 2007). CP can result from early brain developmental problems, during the prenatal, perinatal, or postnatal periods (Bax et al., 2005; Krigger, 2006; Rosenbaum et al., 2007). The most common causes are related to complications from premature birth (e.g., asphyxia) and low birth weight (Babcock et al., 2009). The brain of children with CP is immature, vulnerable, and prone to intraparenchymal, or intraventricular, bleeds or periventricular white-matter abnormalities (Peralta-Carcelen et al., 2009). In addition, in regards to infants, the causes may be related to infection, placental abnormalities, restricted intrauterine growth, and traumatic brain injury (Aisen et al., 2011).

The characteristics of this disorder are determined by the type of brain lesion and the gestational time in which it occurs (e.g., periventricular leukomalacia), the nature of the body impairment (e.g., spasticity, dyskinesia) with different pathophysiological features, and the part of the body impaired or topographical description of CP (Gorter et al., 2004; Graham et al., 2016). Therefore, CP can also be classified into two main physiological groups: pyramidal lesions, which are frequently related to spastic hypertonic, deep-tendon reflexes, overflow reflexes and extensor plantar response; and extrapyramidal lesions, which may be related to choreoathetosis and dyskinesias, abnormal postural control, and coordination deficits (Rosenbaum et al., 2007; Pakula et al., 2009). Finally, when referring to body impairment, another type of motor deficit, the following types are included: dyskinetic, ataxic, spastic and mixed (Straub and Obrzut, 2009). The dyskinetic type is characterized by slowed, uncontrolled, and writhing movements; in some cases, drool and grimace may be observed. In the ataxic type, difficulties in coordination and balance are observed and may be expressed in gait difficulties and fine motor problems. The most common type is the spastic type, which is characterized by increased deep tendon reflexes and muscle tone, tremors, muscle weakness, and gait disturbances (Sankar and Mundkur, 2005; Yeargin-Allsopp et al., 2008). A common impairment associated with the spastic type is dysarthria, a set of oromotor problems with presence of drooling and swallowing difficulties. Finally, 30% of the cases of children with CP show a mixed clinical picture, which refers to the combination of different types of motor deficits (Sankar and Mundkur, 2005). Hence, according to this topographic classification, CP can be divided into two major categories: unilateral (one side of the body is totally or partially affected) and bilateral (the two sides of the body are totally or partially affected). Unilateral CP is comprised of monoplegia (one limb is affected, and often the lower limb) and hemiplegia (upper and lower unilateral extremity impairment). Bilateral CP includes diplegia (all limbs are impaired with the lower limbs more affected than the upper limbs), tetraplegia (upper and lower unilateral limbs affected with a third limb affected in the other side of the body, mostly the lower), and quadriplegia (all four limbs and the trunk are impaired) (Rosenbaum et al., 2007; Bialik and Givon, 2009; Pakula et al., 2009; Graham et al., 2016).

CP is also accompanied by a collection of impairments that can include cognitive, sensory, communication, and perceptual deficits, as well as a lack of emotional, behavioral, and social competences (Odding et al., 2006; Parkes et al., 2009). These impairments echo in the activities of daily life and in the learning process (Mutsaarts et al., 2006) with repercussions in the assessment of children due to, for example, their communication impairments. Importantly, children with CP are especially prone to display working memory and EF deficits (Jenks et al., 2009a; Pueyo et al., 2009), which may help to explain some of their social and learning problems (Bottcher et al., 2009; Di Lieto et al., 2017). In this regard, some authors focused on understanding the relationships between the topography of the lesion (e.g., unilateral) and the motor type (e.g., spastic) classification and the EF deficits (e.g., Di Lieto et al., 2017). For example, Pueyo et al. (2009) analyzed EF performance of children with spastic, dyskinetic, and mixed CP and found EF deficits on preservation and abstract reasoning. Moreover, the systematic review by Weierink et al. (2013) analyzed studies comprising unilateral lesions (left and right), bilateral lesions, and spastic CP motor type (no studies examined dyskinetic, ataxic or mixed motor type). General findings indicated that: (1) attention, inhibitory, and shifting EF skills are frequently impaired (e.g., Kolk and Talvik, 2000; Korkman et al., 2008; Bottcher et al., 2009); (2) bilateral lesions are more related with lower EF performance than unilateral lesions (e.g., Pirila et al., 2011). Nevertheless, regarding unilateral lesions, results suggest that right unilateral CP children performed poorly in EF (i.e., selective auditory, attention and vigilance, shifting and maintaining a complex set involving inhibition) compared to left unilateral CP children (Kolk and Talvik, 2000). This last result is consistent with those by Bodimeade et al. (2013).

### Purpose of the study

A recent study has shown that EFs in children with CP remain stable throughout the developmental span when no intervention is conducted (Piovesana et al., 2015). Moreover, Zelazo et al. (2016) found that the EFs are highly plastic, i.e., subject to changes during the development. These changes are connected to the experience that the different environments may offer to the individual (e.g., school context). Specifically focusing on CP, Graham et al. (2016) highlight that neuroplasticity is a crucial ally to the rehabilitation processes. Additionally, these authors also suggest that the rehabilitation process should be set up to be as precocious as possible in order to take into account the sensitive periods of brain development (Graham et al., 2016). Therefore, an accurate evaluation of the EFs allows rehabilitation therapists to design intervention plans that are adjusted to the needs of the patient and monitor the process of rehabilitation (Zelazo et al., 2016).

This observation supports the need to systematize the body of knowledge of measures to assess EFs in this population. To the authors' best knowledge, no systematic reviews focused on the evaluation of EFs in children and adolescent with CP have been conducted. Therefore, the goal of this systematic review was to understand which EFs measures were used to evaluate children and adolescents with CP under 21 years old. It should be noted that the current study was focused on investigations examining the executive function and not on the executive functioning.

The findings are expected to contribute to clinical practice and future research. The methods used in the present systematic review conform to current Cochrane recommendations (Higgins and Green, 2008) and with Preferred Reporting Items for Systematic Reviews and Meta-Analyses (PRISMA) guidelines (Moher, 2009). Finally, this study did not include human participation; therefore, no ethical approval was required.

## Method

### Search strategy

The initial literature searches were conducted in four databases by SL at the WebScience, Scopus, PubMed, and Psycinfo in November, 2016. All studies published until 2016 were included in the search. The key terms used in the search were “cerebral palsy” AND “executive function” see Table 1. Regarding that EF has become a divergent construct (e.g., function vs. functioning), the search was directed for “Executive Function” within the target population. The date range and the language of publication were used as search limits for publication selection. For the data extraction, Cochrane's recommendations (Higgins and Green, 2008) and the protocol based on the recommendations of the PRISMA statement were followed (Moher, 2009). Accordingly, in the current study, the search strategy of the literature was conducted by two independent reviewers.

**Table 1:** Results obtained in the data search conducted in the four databases (WebScience, Scopus, PubMed, and Psycinfo) with the key-terms combination (“executive function” AND “cerebral palsy”).

**Database**	**Combination**	**Hits per strategy**	**Unique Studies**	**Relevant Studies**	**Included Studies**
WebScience	“executive function” AND “cerebral palsy”	63	50	32	11
Scopus		113	58	26	2
PubMed		48	15	4	0
Psycinfo		625	433	25	2
Total		849	556	87	15

### Selection criteria

In the Identification and Screening phases, the abstracts were included when they met the inclusion criteria or when SL, regarding the fulfillment of the inclusion criteria a priori defined (see Table 2), was in doubt. For the purposes of the present study, the type of lesion presented by individuals with CP was not a criterion.

**Table 2:** Inclusion and exclusion criteria.

**INCLUSION**	
Participants!	Patient group was diagnosed with CP (at least 50% of the patient group sample);
	Children/adolescents aged age up to 21 years or with a mean minus one standard deviation until 21 years of age.
Method	At least one measure of EF was used in the study;
Publication	The study must have been published in a peer-reviewed journal;
Language	The studies had to be written in Portuguese, Spanish, or English.
**EXCLUSION**	
Article type	Not original research, such as reviews, editorials or commentaries;
	Methodological or technical only;
EF construct distinction	Focused on the executive functioning instead of the executive function. The distinction was possible through:
	(a) searching the key term “executive function;”
	(b) exclusion of papers that only used in their evaluation protocol measures to assess executive functioning and/or when authors of each paper state their object of study the executive functioning.

### Data extraction and quality appraisal

Of the first selection of studies meeting the eligibility criteria, two reviewers (SL and AP) independently assessed all the included publications (eligibility phase). At this stage, the titles and abstracts of all the selected references were checked. For the references that were not agreed upon by both reviewers, the corresponding full texts were retrieved. Following this stage, the items that met the inclusion criteria were included in the study, and the two reviewers reached an agreement regarding their assessments. Furthermore, the references of previous reviews were screened and included in the present study when they were found to be consistent with the purposes of this study.

Thereafter, SL and AP independently extracted the information from the selected publications for the present review. Each study was analyzed separately by SL and AP to minimize any bias and improve the reliability of the findings. The agreement between the reviewers for the inclusion and exclusion of studies was unanimous. Finally, the pool of studies selected to be included in the review was evaluated according to the PRISMA standards (Moher, 2009).

## Results

### Selected studies

#### Details of included studies

After screening the four databases (WebScience, Scopus, PubMed, and Psycinfo), 849 studies were identified. After duplicates (293) were removed, 556 studies remained for title screening. The studies were then excluded by title (266) and abstract (203) when they were found not to be relevant to the purposes of the study. Hereupon, 87 studies were selected as potentially relevant for the review and they were screened for full-text review.

**Figure 1:**
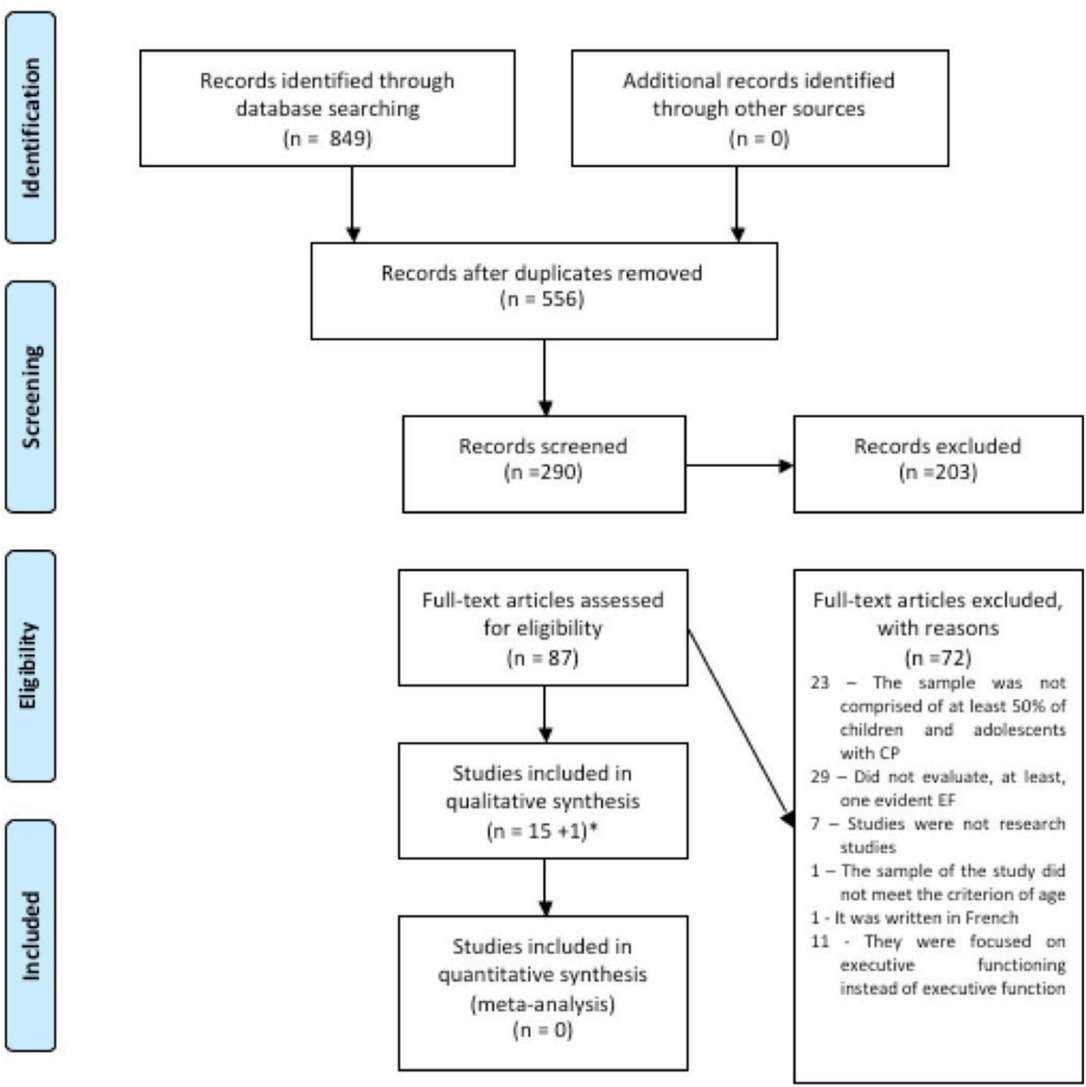
Flow diagram.

Of the 87 studies, 15 were included and 72 were excluded for not meeting the inclusion criteria for this review (see Appendix A in Supplementary Material). Moreover, reference lists of the studies included in this systematic review were checked and one additional study was included, thus, 16 studies were included in total (see Appendix B in Supplementary Material). Reasons for excluding the 72 studies were the following: in 23 of the rejected studies, the sample was not comprised of at least 50% of children and/or adolescents with CP; in 29, EFs were not evaluated; in one, the sample did not meet the age criteria; in 11, the focus was the evaluation of executive functioning; one was written in French; and seven were not research studies (see Appendix A). Therefore, a total of 16 studies were included in this systematic review. Based on PRISMA guidelines (Moher, 2009), the selection process of studies included in the present systematic review is outlined in Figure 1.

Following the selection process, the studies were grouped by journal of origin. The following journals published more than one of the studies included in the present review: *Research in Developmental Disabilities, five* studies (Caillies et al., 2012; Dourado et al., 2013; Gofer-Levi et al., 2014; Li et al., 2014; Ballester-Plané et al., 2016); and *Journal of Child Neurology*, two studies (Jenks et al., 2009b; Pirila et al., 2011). The remaining nine studies were published in different journals (e.g., *Developmental Neuropsychology, British Journal of Educational Psychology*).

Moreover, the studies were developed through five international regions (Europe, the Americas, Oceania, Middle –East, and Asia). Seven studies were developed in the following European countries: Denmark (Bottcher et al., 2009), Finland (Pirila et al., 2011), France (Caillies et al., 2012), Netherlands (Jenks et al., 2009b, 2011), and Spain (Ballester-Plané et al., 2016; Laporta-Hoyos et al., 2017). Three studies were conducted in the following countries in the Americas: Canada (Nadeau et al., 2008), USA (Reilly et al., 2008), and Brazil (Dourado et al., 2013). Three studies were conducted in Oceanian countries: Australia [Bodimeade et al., 2013; Piovesana et al., 2015, 2017]. One study was conducted in the Middle-East, Israel (Gofer-Levi et al., 2014), and one in Asia, China (Li et al., 2014)., One specific study was developed with a sample from five countries and two different continents (Stadskleiv et al., 2014): Europe (Norway, Netherland, Sweden, and Germany) and America (Canada). The sample of studies included in this review are culturally very diverse, but the potential culture bias associated to findings was not addressed by the selected studies. Moreover, no references were made on the adaptations of the instruments and activities developed to the characteristics of children with CP, nor references were provided in regard to cultural aspects about the development of the research and results. Finally, due to the cultural diversity of these investigations, and the potential role of culture for children with CP education and behaviors, readers are encouraged to consider the culture bias likelihood of these studies.

Initially data was organized into two main topics: (i) overview of the characteristics of participants with CP, (ii) instruments/tasks used to evaluate EFs. However, during this process, two new categories emerged (i.e., EFs evaluated and EFs evaluated when a model of EF is adopted). These topics confer more specificity to the main goal of this systematic review.

Appendix C summarizes the studies included in the present review, which are organized chronologically. The form for data extraction included the following: reference, number of participants with CP (percentage of CP in the total sample and information about the age group), characteristics of the sample (gender), objective of the study and design, EFs assessed, instruments or tasks employed, and main results.

Regarding the studies included in this systematic review, 1048 individuals took part in the investigations, in which 698 had been allocated to experimental groups and 350 to control groups. The minimum mean age of participants in these studies was seven years old and the maximum was 25. In this last case, one study was included because of the sample's age standard deviation (*M* = 25.10; *SD* = 12.05; see Appendix C). In two studies (Ballester-Plané et al., 2016; Laporta-Hoyos et al., 2017) the sample was divided into an experimental group and a neuroimaging group.

The majority of the studies used a cross-sectional design (Bottcher et al., 2009; Jenks et al., 2009b, 2011; Pirila et al., 2011; Caillies et al., 2012; Dourado et al., 2013; Stadskleiv et al., 2014), but several studies followed a mixed design (Li et al., 2014; Ballester-Plané et al., 2016; Laporta-Hoyos et al., 2017). Finally, a few studies used other designs such as longitudinal study (Nadeau et al., 2008), exploratory study (Reilly et al., 2008), case control (Gofer-Levi et al., 2014), test-retest (Piovesana et al., 2015), and a randomized-control trial (Piovesana et al., 2017).

Most of the studies included in this review focused on executive dysfunction in patients with CP. The studies focused on assessing participants with CP (Bottcher et al., 2009; Pirila et al., 2011; Piovesana et al., 2015, 2017; e.g., examining whether the EFs in children with CP evolve over time, Piovesana et al., 2015) or focused on comparing participants with CP against typically developing peers (Nadeau et al., 2008; Reilly et al., 2008; Jenks et al., 2009b, 2011; Caillies et al., 2012; Dourado et al., 2013; Li et al., 2014; Stadskleiv et al., 2014; Piovesana et al., 2015; e.g., studies evaluating specific conditions of daily life, such as school performance, Jenks et al., 2009b, 2011 and health issues, Dourado et al., 2013).

### Overview of the characteristics of participants with CP

Through the analysis of the sample characteristics of the 16 studies included in this systematic review, it is possible to observe that specific classifications and previous assessments are present in the majority of the studies (e.g., Motor classification, GMFCS). The motor type and topographic distribution of impairment were referred to in 12 studies (Nadeau et al., 2008; Reilly et al., 2008; Bottcher et al., 2009; Jenks et al., 2009b, 2011; Pirila et al., 2011; Caillies et al., 2012; Bodimeade et al., 2013; Dourado et al., 2013; Gofer-Levi et al., 2014; Li et al., 2014; Piovesana et al., 2015). Only one study classified the sample with a diagnosis of CP without referring to the motor classification (Stadskleiv et al., 2014). Among the selected studies, the characteristics of the sample were diverse. Nine studies classified the motor impairments as Unilateral and Bilateral CP (Bottcher et al., 2009; Jenks et al., 2009b, 2011; Pirila et al., 2011; Bodimeade et al., 2013; Gofer-Levi et al., 2014; Piovesana et al., 2015, 2017; Ballester-Plané et al., 2016), whereas four studies classified them as Hemiplegia and Diplegia (Nadeau et al., 2008; Caillies et al., 2012; Li et al., 2014; Laporta-Hoyos et al., 2017). Some authors added to the topographic distribution of motor impairment information about the motor type (e.g., Spastic, Ataxic) (Bottcher et al., 2009; Jenks et al., 2009b, 2011; Pirila et al., 2011; Caillies et al., 2012; Gofer-Levi et al., 2014; Li et al., 2014), and two studies only mentioned the motor type classification (e.g., Spastic CP) (Reilly et al., 2008; Dourado et al., 2013). Additionally, the classification measures were frequently used to characterize the sample. The Gross Motor Function Classification System (GMFCS) (Palisano et al., 1996) was used in 11 studies (Nadeau et al., 2008; Reilly et al., 2008; Jenks et al., 2009b, 2011; Pirila et al., 2011; Bodimeade et al., 2013; Dourado et al., 2013; Gofer-Levi et al., 2014; Stadskleiv et al., 2014; Piovesana et al., 2015, 2017; Ballester-Plané et al., 2016). Among those 11 studies, the majority of the participants presented a level I in the GMFCS, which represents minor severity (level V represents major severity). The Manual Ability Classification System (MACS) (Eliasson et al., 2006) was used in five studies (Bodimeade et al., 2013; Stadskleiv et al., 2014; Piovesana et al., 2015, 2017; Ballester-Plané et al., 2016), in which the majority of participants displayed a level II. In MACS, level I represents minor severity and level V represents major severity. In two studies, the Communication Function Classification System (CFCS) (Hidecker et al., 2011; Ballester-Plané et al., 2016) was used. Additionally, in one study, where the purpose was to evaluate the EF in children with severe speech and motor problems, the Viking Speech Scale (Pennington et al., 2013) was used. Moreover, intellectual ability was evaluated in nine studies (Jenks et al., 2011; Caillies et al., 2012; Bodimeade et al., 2013; Gofer-Levi et al., 2014; Li et al., 2014; Piovesana et al., 2015, 2017; Ballester-Plané et al., 2016; Laporta-Hoyos et al., 2017), and the WISC was the measure most commonly used in those studies (Caillies et al., 2012; Bodimeade et al., 2013; Li et al., 2014; Piovesana et al., 2015, 2017; Laporta-Hoyos et al., 2017). Also, Raven's Colored Progressive Matrixes was used to assess the non-verbal intelligence performance (Jenks et al., 2011) and global performance (Jenks et al., 2011; Gofer-Levi et al., 2014; Ballester-Plané et al., 2016). The Peabody Picture Vocabulary Test-Revised (Jenks et al., 2011; Ballester-Plané et al., 2016) was used to assess the verbal intelligence and global intelligence performance. In addition, the studies also referred to the associated impairments diagnosed in the samples, including Epilepsy (Bottcher et al., 2009; Jenks et al., 2009b; Bodimeade et al., 2013; Piovesana et al., 2015; Ballester-Plané et al., 2016; Laporta-Hoyos et al., 2017), Intellectual Disability (Piovesana et al., 2015, 2017; Ballester-Plané et al., 2016), Auditory Impairments (Jenks et al., 2009b; Bodimeade et al., 2013; Piovesana et al., 2015, 2017), Visual Impairments (Jenks et al., 2009b; Bodimeade et al., 2013; Piovesana et al., 2015, 2017), Learning Disorder (Bodimeade et al., 2013; Piovesana et al., 2015, 2017), ADHD (Bodimeade et al., 2013; Piovesana et al., 2015, 2017), Autism (Bodimeade et al., 2013; Piovesana et al., 2015, 2017), Anxiety Disorder (Bodimeade et al., 2013; Piovesana et al., 2017), and Other Diseases (Bodimeade et al., 2013; Piovesana et al., 2017). Finally, in six of the studies, the schooling of participants was mentioned (Nadeau et al., 2008; Bottcher et al., 2009; Jenks et al., 2009b, 2011; Ballester-Plané et al., 2016; Laporta-Hoyos et al., 2017). The majority of children and adolescents with CP that participated in these studies attended mainstream schools.

### Instruments/tasks used to evaluate executive functions

In the studies included in this systematic review, the instruments selected to assess EFs were very diverse (e.g., TEA-Ch, Bottcher et al., 2009, BAC, Stadskleiv et al., 2014, the inhibitory ability task, Li et al., 2014; see Appendix C for full list of instruments). Despite the diversity of instruments found, the Delis-Kaplan Executive Function System (D-KEFS) (Delis et al., 2001) was the only instrument used in more than one of the included studies (Bodimeade et al., 2013; Piovesana et al., 2015, 2017). This instrument is comprised of nine tests that evaluate aspects of EFs in the verbal and spatial domains. These tests assess the integrity of the frontal system of the brain (i.e., Trail Making Test, Verbal Fluency Test, Design Fluency Test, Color-Word Interference Test, Sorting Test, 20 Questions Test, Word Context Test, Tower Test, and the Proverb Test). Of these tests, only four were used in the selected studies: Color-Word Interference Test, Verbal Fluency Test, Trail Making Test, and the Tower Test. Participants in these studies were children and adolescents with unilateral CP (right, left or non-specified).

When analyzing the method section of the studies, readers may learn that the majority of the studies only used one task to assess each EF (Nadeau et al., 2008; Bottcher et al., 2009; Jenks et al., 2011; Pirila et al., 2011; Gofer-Levi et al., 2014; Li et al., 2014; Stadskleiv et al., 2014; Ballester-Plané et al., 2016; Laporta-Hoyos et al., 2017; e.g., Inhibition: The inhibitory ability task). A few studies used more than one measure to assess each EF (Reilly et al., 2008; Caillies et al., 2012; Dourado et al., 2013; e.g., Dual-task; Visual working memory task; COP Movement in Single and dual task conditions, Reilly et al., 2008), yet four studies selected more than one measure for some EFs. However, some studies used simultaneously one task and multiple measures to assess different EFs (Jenks et al., 2009b; Bodimeade et al., 2013; Piovesana et al., 2015, 2017). Complementarily, three studies (Bottcher et al., 2009; Piovesana et al., 2015, 2017) included a report measure of EF - BRIEF (Behavior Rating Inventory of Executive Function) in their assessment protocol. Findings do not offer evidence on the fit between the selection of the instrument/task and the motor classification of the participants (e.g., Wisconsin Card Sorting Test was used both with Right Hemiplegia and Diplegia participants), except for the studies by Bodimeade et al. (2013) and Piovesana et al. (2015, 2017) which selected D-KEFS to evaluate EF in included participants with unilateral CP.

Concerning the electrophysiological and anatomical imaging techniques, only two studies included in this systematic review included these measures (Ballester-Plané et al., 2016; Laporta-Hoyos et al., 2017). In these studies, the neuroimaging was collected using MRI acquisition, and it was aimed to complement the instruments, questionnaires, or task performance of children and adolescents with CP.

### Executive functions evaluated

A transversal analysis of the studies showed the following: (i) Attention was the EF evaluated in most studies (Reilly et al., 2008; Bottcher et al., 2009; Pirila et al., 2011; Bodimeade et al., 2013; Piovesana et al., 2015, 2017; Laporta-Hoyos et al., 2017); (ii) Cognitive Flexibility was the second most evaluated EF of the included studies (Nadeau et al., 2008; Bodimeade et al., 2013; Gofer-Levi et al., 2014; Ballester-Plané et al., 2016; Laporta-Hoyos et al., 2017; Piovesana et al., 2017), (iii) Inhibition and Shifting (Bottcher et al., 2009; Jenks et al., 2009b, 2011; Caillies et al., 2012; Dourado et al., 2013) and Working Memory were both assessed in five studies (Jenks et al., 2009b, 2011; Caillies et al., 2012; Dourado et al., 2013; Piovesana et al., 2015), and (iv) Updating was assessed in three studies (Jenks et al., 2009b, 2011; Li et al., 2014). Finally, five studies assessed only one EF (Nadeau et al., 2008; Reilly et al., 2008; Pirila et al., 2011; Dourado et al., 2013; Gofer-Levi et al., 2014). Of these studies, Attention (Reilly et al., 2008; Pirila et al., 2011) and Cognitive Flexibility (Nadeau et al., 2008; Gofer-Levi et al., 2014) were assessed in two of them, while Working Memory, defined as an EF, was assessed in one (Dourado et al., 2013). Finally, Risk Taking was assessed in the other two studies (Ballester-Plané et al., 2016; Laporta-Hoyos et al., 2017); this specific function was found to be associated with decision making. Regarding attention, the selected studies focused on this skill as follows: as a set of skills (attentional control, Bodimeade et al., 2013; Piovesana et al., 2015, 2017; Laporta-Hoyos et al., 2017, executive attention, Reilly et al., 2008), and types of attention (sustained, divided) (Bottcher et al., 2009; Pirila et al., 2011).

Additionally, the majority of the studies included in this systematic review showed that children and adolescents with CP show EF deficits. Specifically, executive dysfunction may be present in a specific EF (e.g., Cognitive Flexibility, Gofer-Levi et al., 2014) or, concurrently, in several EFs (Jenks et al., 2009b, 2011; Pirila et al., 2011). The 16 selected studies investigated the performance of children in school and activities of daily life. The majority of the studies investigated the level of executive dysfunction in children and/or adolescents with CP (Nadeau et al., 2008; Reilly et al., 2008; Bottcher et al., 2009; Jenks et al., 2009b, 2011; Pirila et al., 2011; Caillies et al., 2012; Bodimeade et al., 2013; Dourado et al., 2013; Gofer-Levi et al., 2014; Li et al., 2014; Stadskleiv et al., 2014). In addition, (Piovesana et al., 2015, 2017), investigated how EFs evolve in this specific population. The first study, a test-retest reliability study, (Piovesana et al., 2015) aimed to analyze if the EF performance changes after 20 weeks without EF training. The results did not show any changes in EFs performance during the 20-week period. The second study, a randomized controlled trial, was conducted for 20 weeks with an interactive web-based multi-modal training intervention (Piovesana et al., 2017). Authors observed that after the 20 weeks of the Mitii™ program (Move-It-To-Improve-It), the EFs remained stable over time when no motor intervention was conducted (Piovesana et al., 2017). Also, considering the selected studies, only the studies by Piovesana et al. (2015) assessed the test-retest reliability of measures used to evaluate the EFs and the efficacy of an intervention program designed to improve EF performance (Piovesana et al., 2017).

### Executive functions evaluated when a model of executive functions is adopted

Six studies included in the current review mentioned following a theoretical model of EF (Jenks et al., 2009b, 2011; Bodimeade et al., 2013; Li et al., 2014; Piovesana et al., 2015, 2017). Three studies declared to follow Anderson's Model of EFs (Anderson, 2002) to assess EFs (Bodimeade et al., 2013; Piovesana et al., 2015, 2017). In the first study, Bodimeade et al. (2013) assessed Cognitive Flexibility with the Digit Span Backward sub-test from the Wechsler Intelligence Scale for Children, 4th edition (WISC-IV), the Trail Making Test, and the Verbal Fluency Test from the D-KEFS. To evaluate Goal Setting, the authors used the Color-Word Interference Test from the D-KEFS. Information Processing was evaluated with the Verbal Fluency Test and the Tower Test from the D-KEFS, together with the Rey-Osterrieth Complex Figure Test. Finally, Attentional Control was assessed using the Code Transmission Test, the Symbol Search and Cancellation from WISC-IV, the Trail Making Test, the Verbal Fluency Test, and the Color-Word Interference Test from D-KEFS. In the other two studies, Piovesana and colleagues (Piovesana et al., 2015, 2017) evaluated the EFs with the following tasks: Cognitive Flexibility was evaluated with the Color-Word Interference Test and the Trail Making Test from D-KEFS and the Digit Span Backward sub-test from the WISC-IV; Goal Setting was evaluated with the Tower Test from the D-KEFS; Attentional Control was assessed using the Color-Word Interference Test (Inhibition Condition) from D-KEFS; and Information Processing was evaluated with the Code and Symbol Search sub-test from WISC-IV. These studies also included the assessment of EF using BRIEF (Piovesana et al., 2015, 2017).

The other three studies included in this review declared to follow the model of EFs by Miyake et al.'s (2000a), and they evaluated Inhibition, Shifting, and Updating (Jenks et al., 2009b, 2011; Li et al., 2014). In the first study, Jenks and colleagues (Jenks et al., 2009b) used the shifting-naming task and the inhibition-naming task to study Shifting and Inhibition respectively (i.e., the van der Sluis, de Jong, and van der Leij inhibition, and shifting task). The Updating function was evaluated using the Backwards Digits task. In the second study, Jenks and colleagues (Jenks et al., 2011) added to the assessment Working Memory function, measured using the Knox blocks, the Both Digit Recall, and the Word Recall. Lastly, the third study (Li et al., 2014) used the inhibitory ability, the information updating task, and the attention-shifting task to assess Inhibition, Updating, and Shifting respectively.

## Discussion

The synthesis of knowledge conducted in this systematic review aimed to deepen the understanding of how EFs are being assessed in children and adolescents with CP. Regarding this aim, the results of the present systematic review highlight the diversity of tests used to assess EFs in CP. Moreover, the sample of studies included did not acknowledge why and how well the tests used match the specificities of the children with this clinical condition. For this reason, the instruments used in the studies organized the discussion section.

The D-KEFS (Delis et al., 2001) was the only instrument used in more than one study (Bodimeade et al., 2013; Piovesana et al., 2015, 2017). In fact, this composition of nine different tests embraces the assessment of more than one EF, providing a more global evaluation of EFs overall. The majority of the studies included in the present study used standardized tests. This methodological option is likely to contribute to minimize the impact of “task impurity” reported by Miyake et al. (2000b). Moreover, most of the studies included in this review used only one test to evaluate each EF (Nadeau et al., 2008; Jenks et al., 2009b, 2011; Pirila et al., 2011; Li et al., 2014; Stadskleiv et al., 2014). That option could be related to the limited availability of a diverse set of standard measures to assess EFs. In fact, authors may have difficulties to find distinct standardized measures to assess EFs on an individual level; besides, to the present authors' knowledge, none of the measures used were validated to this specific population (children or adolescents with CP), nor adaptations to the clinical specificities of this population (e.g., due to language impairment or motor speed) were reported. Finally, only three studies used more than one test to evaluate each EF (Reilly et al., 2008; Dourado et al., 2013; Gofer-Levi et al., 2014). The latter is referred in the literature as a good practice to minimize “task impurity”, and possible ceiling effects (Miyake et al., 2000b; Hughes and Graham, 2002; Best and Miller, 2010), despite each measure having its own “impurity” problems. Recently, literature (e.g., Di Lieto et al., 2017) suggested the need to use non-verbal standardized tests or sub-tests (e.g., Backward Memory task from Leiter-R; WCST) with high reliability to minimize “task impurity” problems and motor and/or language impairment bias.

The studies included their lack of information regarding the nature and the application of the measures. For example, the psychometric properties of the measures were not reported, and the information on whether the characteristics of the measures (e.g., time limits) were considered against the characteristics of this population (e.g., type of motor impairment) were not offered to readers. As mentioned in the results section of the present study, only the study by Piovesana et al. (2015) assessed the test-retest reliability in this specific population. Reliable information on the measures and methodology followed is important for the practice of clinicians and researchers: the research published is expected to help further improve the quality of practice and future investigations. Future researchers in this area may want to consider designing new tasks and instruments to assess EFs and evaluate their reliability using a test-retest scope.

Another relevant result that emerged from this systematic review is that there are particular EFs that are evaluated more often. Attention was the EF evaluated in most studies, followed by Cognitive Flexibility. Moreover, in the studies that evaluated only one EF, Attention and Cognitive Flexibility were often the EFs to be assessed.

The present study has stressed the existence of impairments related to different EFs in children and adolescents with CP. Moreover, the findings also draw attention to the pervasiveness of these impairments in many life domains. Specifically, some studies investigated how EFs are related to clinical conditions (e.g., postural control, Reilly et al., 2008, speech and motor impairment, Stadskleiv et al., 2014), or academic skills (e.g., arithmetic ability, word-problem solving, and reading problems, Jenks et al., 2009b, 2011). Literature reports that children with CP are prone to develop learning disabilities (Muter, 1994; Michel et al., 2011); however, this predisposition is not completely determined by cognitive deficits. Young children with normative cognitive levels are also prone to present specific learning difficulties (e.g., mathematics, reading; Frampton et al., 1998). Executive dysfunction, namely in Attention and Cognitive Flexibility, could help explain the increased learning problems displayed by children and adolescents with CP (Bottcher et al., 2009; Bodimeade et al., 2013). Hence, specific training of the EFs of children with CP could promote their school success. For example, the studies by Jenks et al. (2009b); Jenks et al. (2011) draw the attention of educators and researchers to the need of further the knowledge regarding the learning difficulties of children and adolescents with CP. Findings from future studies in this area could help with the design of special educational curricula so that the curricula may fit the needs of this population. Moreover, these studies could also suggest key aspects for promoting inclusive school environments in the mainstream schools for children and adolescents with CP.

In the same line of reasoning, studies examining Attention (Reilly et al., 2008; Bottcher et al., 2009; Pirila et al., 2011) and Working Memory (Caillies et al., 2012) add important information about the performance of this population in school. These findings show not only the performance of children and adolescents with CP but also the present strategies and methods that can stimulate their competencies and increase their academic achievement. In fact, Haan (2013) highlights that several training studies show close relationships between training EFs and improvements in academic achievement.

Another avenue for future research may be drawn from the study by Piovesana et al. (2015), which concluded that EFs in individuals with CP are likely to remain stable over time (Piovesana et al., 2015). After the 20 weeks of Mitii™ intervention, the participants showed no significant improvement in their EF performance (Piovesana et al., 2017). This finding could be related to the nature of the intervention, which was more focused on the motor training than on specific EF training. The authors (Piovesana et al., 2017) suggested that more directed EF interventions (e.g., Cogmed®) should be designed. There is a need to conduct research aimed to develop effective intervention programs targeting the population with CP. These studies should focus on the stimulation of deficient EFs (e.g., randomized-control trials), as well as the identification of individual differences in terms of EFs (e.g., longitudinal studies). Future research could consider implementing neuro-rehabilitation processes at the early stages of development with new and promising methods (e.g., promotion of self-regulatory competences, Rosário et al., 2016 serious games, Susi et al., 2007) for the promotion of developing EFs. Importantly, therapists, parents/caregivers, and teachers, could be provided with adequate training and support to ensure the transfer of the intervention gains to the daily contexts (Dawson and Guare, 2013). The early diagnosis and intervention would also benefit from the potential of neuroplasticity (Aisen et al., 2011; Graham et al., 2016). The studies included in the present sample only evaluated children attending school. Despite the importance of school in the stimulation of the EFs (e.g., embed EF skills and strategies training in the curricula), an evaluation and intervention at an early age (early childhood) is expected to provide extra opportunities to enhance the EFs development (Dawson and Guare, 2013; Graham et al., 2016; Zelazo et al., 2016). This early intervention is likely to prepare children for the school challenges and prevent deep learning problems.

Besides reporting the importance of cognitive training, literature stress other alternatives to address EF deficits; for example, the importance of setting physical and social environment modifications, such as wheelchair ramps in the schools or teaching augmentative and alternative language to the whole class in which a child or adolescent with CP in integrated in. These educational interventions are expected to facilitate the engagement of children with CP in school activities and in daily life activities, which will favor their EF development (Dawson and Guare, 2013).

In sum, this systematic review adds to the literature as it provides an updated summary of the measures used to evaluate EFs in children and adolescents with CP. One important finding, which comprises simultaneously a limitation of the current review, concerns the fact that the studies examined did not mention whether EF tasks or instruments used to assess the EF were modified or adapted to fit the characteristics of the clinical condition. This relevant information would have allowed this review to report to clinicians and practitioners the best practices suited to the different characteristics of CP. Future studies could consider including complete information about the assessment process and the adaptations made to fit children with CP needs.

To guarantee the quality of the assessment of the studies selected for this review, the authors conformed to the Cochrane's guidelines (Higgins and Green, 2008) and PRISMA statement (Moher, 2009). However, despite the search strategy adopted being a thorough one, the authors cannot guarantee that all data and important results were recovered; for example, the search did not include unpublished material or other material such as “gray literature.” In fact, studies with non-significant results are scarcely submitted or accepted for publication. For this reason, the published literature may be unrepresentative of the entirety of EF studies. Moreover, a publication bias in the literature should be acknowledged. Another potential source of selection bias in this assessment may be the language bias, as only studies published in English, Spanish, or Portuguese were selected. However, the impact of excluding studies published in languages other than English has been shown to have little effect on short treatment estimates.

Regarding the present study, the authors hope to help researchers build more accurate investigation protocols and provide practitioners with robust guidelines for future interventions. Future generations may benefit from the findings emerging from further studies on EF that focus on educational, health, and professional challenges (Diamond, 2013). This effort becomes more necessary in order to understand the potential of EF training in children and adolescents with disabilities, such as CP. This will help improve the quality of life and development of these individuals. For example, bridging the educational gap between some children and adolescent with CP and children with typical development could increase inclusion and acceptance of the youth with CP.

## Author contributions

SL was responsible for the literature search and data extraction. SL and AP were responsible for the blinding literature search and the data extraction. PM and AP oversaw data interpretation and technical guidance. PR, EC, and AS made important intellectual contribution in research design and manuscript revision.

### Conflict of interest statement

The authors declare that the research was conducted in the absence of any commercial or financial relationships that could be construed as a potential conflict of interest.
