# Supplementary material for: How Executive Functions Are Evaluated in Children and Adolescents with Cerebral Palsy? A Systematic Review
Source: Front Psychol. 2018 Feb 6;9:21. doi: 10.3389/fpsyg.2018.00021 (PMC5808176; doi:10.3389/fpsyg.2018.00021)
Supplement: Supplementary file 1 [file Table1.docx]

**Appendix A**. Summary of reasons for exclusion from systematic review

| **Criterion 1. The sample was not comprised of at least 50% of children and adolescents with CP** |
| --- |
|  |
| Aarnoudse-Moens, Cornelieke S.H., Hugo J. Duivenvoorden, Nynke Weisglas-Kuperus, Johannes B Van Goudoever, and Jaap Oosterlaan. “The Profile of Executive Function in Very Preterm Children at 4 to 12 Years.” Developmental Medicine & Child Neurology 54, no. 3 (November 29, 2011): 247–253. doi:10.1111/j.1469-8749.2011.04150.x. |
| Abily-Donval, Lénaïg, Gaëlle Pinto-Cardoso, Alexandra Chadie, Anne-Marie Guerrot, Stéphanie Torre, Stéphane Rondeau, and Stéphane Marret. “Comparison in Outcomes at Two-Years of Age of Very Preterm Infants Born in 2000, 2005 and 2010.” Edited by Pierre Gressens. PLOS ONE 10, no. 2 (February 6, 2015): e0114567. doi:10.1371/journal.pone.0114567.  Belmonti, Vittorio, Alain Berthoz, Giovanni Cioni, Simona Fiori, and Andrea Guzzetta. “Navigation Strategies as Revealed by Error Patterns on the Magic Carpet Test in Children with Cerebral Palsy.” Frontiers in Psychology 6 (July 8, 2015). doi:10.3389/fpsyg.2015.00880. |
| Böhm, Birgitta, Aiko Lundequist, and Ann-Charlotte Smedler. “Visual-Motor and Executive Functions in Children Born Preterm: The Bender Visual Motor Gestalt Test Revisited.” Scandinavian Journal of Psychology (March 22, 2010): no–no. doi:10.1111/j.1467-9450.2010.00818.x. |
| Boyd, Roslyn N., Emmah Baque, Adina Piovesana, Stephanie Ross, Jenny Ziviani, Leanne Sakzewski, Lee Barber, et al. “Mitii™ ABI: Study Protocol of a Randomised Controlled Trial of a Web-Based Multi-Modal Training Program for Children and Adolescents with an Acquired Brain Injury (ABI).” BMC Neurology 15, no. 1 (August 19, 2015). doi:10.1186/s12883-015-0381-6. |
| Brouwer, A.J., C. van Stam, M. Uniken Venema, C. Koopman, F. Groenendaal, and L.S. de Vries. “Cognitive and Neurological Outcome at the Age of 5–8 Years of Preterm Infants with Post-Hemorrhagic Ventricular Dilatation Requiring Neurosurgical Intervention.” Neonatology 101, no. 3 (2012): 210–216. doi:10.1159/000331797. |
| Buuren Van, Lenny M, Niek E van der Aa, Harmke C Dekker, R Jeroen Vermeulen, Onno van Nieuwenhuizen, Monique M J van Schooneveld, and Linda S de Vries. “Cognitive Outcome in Childhood after Unilateral Perinatal Brain Injury.” Developmental Medicine & Child Neurology 55, no. 10 (June 13, 2013): 934–940. doi:10.1111/dmcn.12187. |
| Chen M.D., Tsai H.Y., Wang C.C., Wuang Y.P. “The Effectiveness of Racket-Sport Intervention on Visual Perception and Executive Functions in Children with Mild Intellectual Disabilities and Borderline Intellectual Functioning.” Neuropsychiatric Disease and Treatment (September 2015): 2287. doi:10.2147/ndt.s89083.  Harris, DL, PJ Weston, JM Alsweiler, B Thompson, T Wouldes, G Chase, Y Jiang, G Gamble, and JE Harding. “O-104 Two Year Outcomes Of Children Treated With Dextrose Gel For Neonatal Hypoglycaemia: Follow Up Of A Randomised Trial.” Archives of Disease in Childhood 99, no. Suppl 2 (October 2014): A64.2–A65. doi:10.1136/archdischild-2014-307384.171. |
| Kallankari, Hanna, Tuula Kaukola, Päivi Olsén, Marja Ojaniemi, and Mikko Hallman. “Very Preterm Birth and Foetal Growth Restriction Are Associated with Specific Cognitive Deficits in Children Attending Mainstream School.” Acta Paediatrica 104, no. 1 (October 7, 2014): 84–90. doi:10.1111/apa.12811. |
| Korkman, Marit, Kaija Mikkola, Niina Ritari, Viena Tommiska, Teija Salokorpi, Leena Haataja, Outi Tammela, Leena Pääkkönen, Päivi Olsén, and Vineta Fellman. “Neurocognitive Test Profiles of Extremely Low Birth Weight Five-Year-Old Children Differ According to Neuromotor Status.” Developmental Neuropsychology 33, no. 5 (August 28, 2008): 637–655. doi:10.1080/87565640802254471. |
| Marlow, N., E. M. Hennessy, M. A. Bracewell, and D. Wolke. “Motor and Executive Function at 6 Years of Age After Extremely Preterm Birth.” Pediatrics 120, no. 4 (October 1, 2007): 793–804. doi:10.1542/peds.2007-0440. |
| Martinez-Biarge, Miriam, Victoria C. Jowett, Frances M. Cowan, and Courtney J. Wusthoff. “Neurodevelopmental Outcome in Children with Congenital Heart Disease.” Seminars in Fetal and Neonatal Medicine 18, no. 5 (October 2013): 279–285. doi:10.1016/j.siny.2013.04.006. |
| Pritchard, Verena E., and Lianne J. Woodward. “Preschool Executive Control on the Shape School Task: Measurement Considerations and Utility.” Psychological Assessment 23, no. 1 (2011): 31–43. doi:10.1037/a0021095. |
| Shankaran, Seetha, Athina Pappas, Scott A. McDonald, Betty R. Vohr, Susan R. Hintz, Kimberly Yolton, Kathryn E. Gustafson, et al. “Childhood Outcomes after Hypothermia for Neonatal Encephalopathy.” New England Journal of Medicine 366, no. 22 (May 31, 2012): 2085–2092. doi:10.1056/nejmoa1112066. |
| Sherlock, R.L., P.J. Anderson, and L.W. Doyle. “Neurodevelopmental Sequelae of Intraventricular Haemorrhage at 8 Years of Age in a Regional Cohort of ELBW/very Preterm Infants.” Early Human Development 81, no. 11 (November 2005): 909–916. doi:10.1016/j.earlhumdev.2005.07.007. |
| Skranes, Jon, Karianne Indredavik Evensen, Gro C. Løhaugen, Marit Martinussen, Siri Kulseng, Gunnar Myhr, Torstein Vik, and Ann–Mari Brubakk. “Abnormal Cerebral MRI Findings and Neuroimpairments in Very Low Birth Weight (VLBW) Adolescents.” European Journal of Paediatric Neurology 12, no. 4 (July 2008): 273–283. doi:10.1016/j.ejpn.2007.08.008. |
| Steinhorn, Rachel, Christopher McPherson, Peter J. Anderson, Jeffrey Neil, Lex W. Doyle, and Terrie Inder. “Neonatal Morphine Exposure in Very Preterm Infants—Cerebral Development and Outcomes.” The Journal of Pediatrics 166, no. 5 (May 2015): 1200–1207.e4. doi:10.1016/j.jpeds.2015.02.012. |
| Thompson, Deanne K., Katherine J. Lee, Gary F. Egan, Simon K. Warfield, Lex W. Doyle, Peter J. Anderson, and Terrie E. Inder. “Regional White Matter Microstructure in Very Preterm Infants: Predictors and 7 Year Outcomes.” Cortex 52 (March 2014): 60–74. doi:10.1016/j.cortex.2013.11.010. |
| Vandborg, Pernille Kure, Bo Moelholm Hansen, Gorm Greisen, Rene Mathiasen, Frederikke Kasper, and Finn Ebbesen. “Follow-up of Extreme Neonatal Hyperbilirubinaemia in 5- to 10-Year-Old Children: a Danish Population-Based Study.” Developmental Medicine & Child Neurology 57, no. 4 (October 29, 2014): 378–384. doi:10.1111/dmcn.12603. |
| Wilson, Kathryn R., Jacobus Donders, and Loan Nguyen. “Self and Parent Ratings of Executive Functioning after Adolescent Traumatic Brain Injury.” Rehabilitation Psychology 56, no. 2 (2011): 100–106. doi:10.1037/a0023446. |
| Yeates, Keith Owen, Erin D. Bigler, Maureen Dennis, Cynthia A. Gerhardt, Kenneth H. Rubin, Terry Stancin, H. Gerry Taylor, and Kathryn Vannatta. “Social Outcomes in Childhood Brain Disorder: A Heuristic Integration of Social Neuroscience and Developmental Psychology.” Psychological Bulletin 133, no. 3 (2007): 535–556. doi:10.1037/0033-2909.133.3.535.  Young, Julia M., Benjamin R. Morgan, Tamara L. Powell, Aideen M. Moore, Hilary E.A. Whyte, Mary Lou Smith, and Margot J. Taylor. “Associations of Perinatal Clinical and Magnetic Resonance Imaging Measures with Developmental Outcomes in Children Born Very Preterm.” The Journal of Pediatrics 170 (March 2016): 90–96. doi:10.1016/j.jpeds.2015.11.044. |
|  |
| **Criterion 2. Did not evaluate, at least, one evident EF** |
|  |
| Belmonti, V., Fiori, S., Guzzetta, A., Cioni, G., Berthoz, A. “Cognitive strategies for locomotor navigation in normal development and cerebral palsy”. Developmental Medicine & Child Neurology. Wiley-Blackwell, 17 (2015, Feb): 31–6. doi:10.1111/dmcn.12685.  Bleyenheuft, Yannick, Laurence Dricot, Nathalie Gilis, Hsing-Ching Kuo, Cécile Grandin, Corinne Bleyenheuft, Andrew M. Gordon, and Kathleen M. Friel. “Capturing Neuroplastic Changes after Bimanual Intensive Rehabilitation in Children with Unilateral Spastic Cerebral Palsy: A Combined DTI, TMS and fMRI Pilot Study.” Research in Developmental Disabilities 43–44 (August 2015): 136–149. doi:10.1016/j.ridd.2015.06.014.  Choi, Ja Young, Yoon Seong Choi, Dong-wook Rha, and Eun Sook Park. “The Clinical Outcomes of Deep Gray Matter Injury in Children with Cerebral Palsy in Relation with Brain Magnetic Resonance Imaging.” Research in Developmental Disabilities 55 (August 2016): 218–225. doi:10.1016/j.ridd.2016.04.010.  Crajé, Céline, Pauline Aarts, Maria Nijhuis-van der Sanden, and Bert Steenbergen. “Action Planning in Typically and Atypically Developing Children (unilateral Cerebral Palsy).” Research in Developmental Disabilities 31, no. 5 (September 2010): 1039–1046. doi:10.1016/j.ridd.2010.04.007.  Coleman, Andrea, Simona Fiori, Kelly A. Weir, Robert S. Ware, and Roslyn N. Boyd. “Relationship Between Brain Lesion Characteristics and Communication in Preschool Children with Cerebral Palsy.” Research in Developmental Disabilities 58 (November 2016): 55–64. doi:10.1016/j.ridd.2016.08.015.  Damji, Omar, Jamie Keess, and Adam Kirton. “Evaluating Developmental Motor Plasticity with Paired Afferent Stimulation.” Developmental Medicine & Child Neurology 57, no. 6 (January 30, 2015): 548–555. doi:10.1111/dmcn.12704. |
| Forsman, Lea, and Ann-Christin Eliasson. “Strengths and Challenges Faced by School-Aged Children with Unilateral CP Described by the Five To Fifteen Parental Questionnaire.” Developmental Neurorehabilitation (April 2, 2015): 1–9. doi:10.3109/17518423.2015.1017662. |
| Hakkarainen, Elina, Silja Pirilä, Jukka Kaartinen, and Jaap J. van der Meere. “Stimulus Evaluation, Event Preparation, and Motor Action Planning in Young Patients With Mild Spastic Cerebral Palsy.” Journal of Child Neurology 27, no. 4 (April 2012): 465–470. doi:10.1177/0883073811420150. |
| Hakkarainen, E., S. Pirila, J. Kaartinen, and J. J. van der Meere. “Error Detection and Response Adjustment in Youth With Mild Spastic Cerebral Palsy: An Event-Related Brain Potential Study.” Journal of Child Neurology 28, no. 6 (August 16, 2012): 752–757. doi:10.1177/0883073812452786. |
| Hakkarainen, Elina, Silja Pirilä, Jukka Kaartinen, and Jaap J. van der Meere. “Brain State Before Error Making in Young Patients With Mild Spastic Cerebral Palsy.” Journal of Child Neurology 30, no. 11 (October 2015): 1489–1495. doi:10.1177/0883073815571453.  Hastings-Ison, Tandy, Christine Blackburn, Barry Rawicki, Michael Fahey, Pam Simpson, Richard Baker, and Kerr Graham. “Injection Frequency of Botulinum Toxin A for Spastic Equinus: a Randomized Clinical Trial.” Developmental Medicine & Child Neurology 58, no. 7 (November 20, 2015): 750–757. doi:10.1111/dmcn.12962. |
| Hung, Ya-Ching, Jeanne Charles, and Andrew M. Gordon. “Influence of Accuracy Constraints on Bimanual Coordination During a Goal-Directed Task in Children with Hemiplegic Cerebral Palsy.” Experimental Brain Research 201, no. 3 (October 23, 2009): 421–428. doi:10.1007/s00221-009-2049-1.  James, Sarah, Jenny Ziviani, Robert S Ware, and Roslyn N Boyd. “Relationships Between Activities of Daily Living, Upper Limb Function, and Visual Perception in Children and Adolescents with Unilateral Cerebral Palsy.” Developmental Medicine & Child Neurology 57, no. 9 (February 23, 2015): 852–857. doi:10.1111/dmcn.12715.  Kukke, Sahana N., Ana Carolina de Campos, Diane Damiano, Katharine E. Alter, Nicholas Patronas, and Mark Hallett. “Cortical Activation and Inter-Hemispheric Sensorimotor Coherence in Individuals with Arm Dystonia Due to Childhood Stroke.” Clinical Neurophysiology 126, no. 8 (August 2015): 1589–1598. doi:10.1016/j.clinph.2014.11.002.  Lorentzen, Jakob, and Line Petersen. “Twenty Weeks of Home-Based Interactive Training of Children with Cerebral Palsy Improves Functional Abilities.” BMC neurology 15(1) (May, 2015): 1-12. doi:10.1186/isrctn13188513.  Manning, Kathryn Y., Darcy Fehlings, Ronit Mesterman, Jan Willem Gorter, Lauren Switzer, Craig Campbell, and Ravi S. Menon. “Resting State and Diffusion Neuroimaging Predictors of Clinical Improvements Following Constraint-Induced Movement Therapy in Children With Hemiplegic Cerebral Palsy.” Journal of Child Neurology 30, no. 11 (October 2015): 1507–1514. doi:10.1177/0883073815572686.  Marlow, N., Rose, A.S., Rands, C.E., Draper, E.S. “Neuropsychological and educational problems at school age associated with neonatal encephalopathy". Archives of Disease in Childhood-Fetal and Neonatal Edition 90(5) (Feb, 2005): F380-F387. doi:10.1136/adc.2004.067520. |
| Newsham, David, Paul C. Knox, and Richard W. I. Cooke. “Oculomotor Control in Children Who Were Born Very Prematurely.” Investigative Opthalmology & Visual Science 48, no. 6 (June 1, 2007): 2595. doi:10.1167/iovs.06-1425. |
| Olivier, I., C. Baker, J. Cordier, G. Thomann, and V. Nougier. “Cognitive and Motor Aspects of a Coincidence-Timing Task in Cerebral Palsy Children.” Neuroscience Letters 602 (August 2015): 33–37. doi:10.1016/j.neulet.2015.06.043.  Rana, Kamer Singh, Varun Narwal, Lokesh Chauhan, Giriraj Singh, Monica Sharma, and Suneel Chauhan. “Structural and Perfusion Abnormalities of Brain on MRI and Technetium-99m-ECD SPECT in Children With Cerebral Palsy.” Journal of Child Neurology 31, no. 5 (April 2016): 589–592. doi:10.1177/0883073815604224.  Reid, Susan M., Michael R. Ditchfield, Jenny Bracken, and Dinah S. Reddihough. “Relationship Between Characteristics on Magnetic Resonance Imaging and Motor Outcomes in Children with Cerebral Palsy and White Matter Injury.” Research in Developmental Disabilities 45–46 (October 2015): 178–187. doi:10.1016/j.ridd.2015.07.030.  Scheck, Simon M., Jurgen Fripp, Lee Reid, Kerstin Pannek, Simona Fiori, Roslyn N. Boyd, and Stephen E. Rose. “Extent of Altered White Matter in Unilateral and Bilateral Periventricular White Matter Lesions in Children with Unilateral Cerebral Palsy.” Research in Developmental Disabilities 55 (August 2016): 368–376. doi:10.1016/j.ridd.2016.04.007.  Schiariti, V., and L. C. Masse. “Relevant Areas of Functioning in Children With Cerebral Palsy Based on the International Classification of Functioning, Disability and Health Coding System: A Clinical Perspective.” Journal of Child Neurology 30, no. 2 (May 7, 2014): 216–222. doi:10.1177/0883073814533005. |
| Sherwell, Sarah, Susan M. Reid, Dinah S. Reddihough, Jacquie Wrennall, Ben Ong, and Robyn Stargatt. “Measuring Intellectual Ability in Children with Cerebral Palsy: Can We Do Better?” Research in Developmental Disabilities 35, no. 10 (October 2014): 2558–2567. doi:10.1016/j.ridd.2014.06.019. |
| Stasolla, Fabrizio, Rita Damiani, Viviana Perilli, Fiora D’Amico, Alessandro O. Caffò, Anna Stella, Vincenza Albano, Concetta Damato, and Antonia Di Leone. “Computer and Microswitch-Based Programs to Improve Academic Activities by Six Children with Cerebral Palsy.” Research in Developmental Disabilities 45–46 (October 2015): 1–13. doi:10.1016/j.ridd.2015.07.005  Tomita, Hidehito, Yoshiki Fukaya, Yukina Takagi, and Asami Yokozawa. “Effects of Severity of Gross Motor Disability on Anticipatory Postural Adjustments While Standing in Individuals with Bilateral Spastic Cerebral Palsy.” Research in Developmental Disabilities 57 (October 2016): 92–101. doi:10.1016/j.ridd.2016.06.017. |
| Van de Winckel, Ann, Geert Verheyden, Nici Wenderoth, Ron Peeters, Stefan Sunaert, Wim Van Hecke, Paul De Cock, Kaat Desloovere, Maria Eyssen, and Hilde Feys. “Does Somatosensory Discrimination Activate Different Brain Areas in Children with Unilateral Cerebral Palsy Compared to Typically Developing Children? An fMRI Study.” Research in Developmental Disabilities 34, no. 5 (May 2013): 1710–1720. doi:10.1016/j.ridd.2013.02.017.  Vitiello, Damien, Ludmilla Pochon, Davide Malatesta, Olivier Girard, Christopher J. Newman, and Francis Degache. “Walking-Induced Muscle Fatigue Impairs Postural Control in Adolescents with Unilateral Spastic Cerebral Palsy.” Research in Developmental Disabilities 53–54 (June 2016): 11–18. doi:10.1016/j.ridd.2016.01.019.  Zhvansky, E. S., O. N. Tsyshkova, A. A. Grishin, Y. P. Ivanenko, Y. S. Levik, and E. S. Keshishyan. “Characteristics of EMG Activity in Infants with Movement Disorders.” Human Physiology 41, no. 1 (January 2015): 39–46. doi:10.1134/s0362119715010156. |
|  |
| **Criterion 3. Studies were not research studies** |
|  |
| Berthoz, Alain, and Mohamed Zaoui. “New Paradigms and Tests for Evaluating and Remediating Visuospatial Deficits in Children.” Developmental Medicine & Child Neurology 57 (February 17, 2015): 15–20. doi:10.1111/dmcn.12690. |
| Bodimeade, Harriet L, Koa Whittingham, Owen Lloyd, and Roslyn N Boyd. “Executive Functioning in Children with Unilateral Cerebral Palsy: Protocol for a Cross-Sectional Study.” BMJ Open 3, no. 4 (2013): e002500. doi:10.1136/bmjopen-2012-002500. |
| Duerden, Emma G., Margot J. Taylor, and Steven P. Miller. “Brain Development in Infants Born Preterm: Looking Beyond Injury.” Seminars in Pediatric Neurology 20, no. 2 (June 2013): 65–74. doi:10.1016/j.spen.2013.06.007.  Fahey, Michael. “Early Developmental Brain Injury/interference: Moving on from Cerebral Palsy.” Developmental Medicine & Child Neurology 57, no. 7 (June 10, 2015): 681–681. doi:10.1111/dmcn.12776.  Newman, Thomas B, and Michael W Kuzniewicz. “Follow-up of Extreme Neonatal Hyperbilirubinaemia: More Reassuring Results from Denmark.” Developmental Medicine & Child Neurology 57, no. 4 (October 28, 2014): 314–315. doi:10.1111/dmcn.12611. |
| Sansavini, Alessandra, Annalisa Guarini, and Maria Cristina Caselli. “Preterm Birth: Neuropsychological Profiles and Atypical Developmental Pathways.” Developmental Disabilities Research Reviews 17, no. 2 (November 2011): 102–113. doi:10.1002/ddrr.1105.  Straub, Kathryn, and John E. Obrzut. “Effects of Cerebral Palsy on Neuropsychological Function.” Journal of Developmental and Physical Disabilities 21, no. 2 (January 23, 2009): 153–167. doi:10.1007/s10882-009-9130-3. |
|  |
|  |
| **Criterion 4. The sample of the study did not meet the criterion of age** |
|  |
| Husby, Ingrid Marie, Jon Skranes, Alexander Olsen, Ann-Mari Brubakk, and Kari Anne I. Evensen. “Motor Skills at 23years of Age in Young Adults Born Preterm with Very Low Birth Weight.” Early Human Development 89, no. 9 (September 2013): 747–754. doi:10.1016/j.earlhumdev.2013.05.009. |
|  |
| **Criterion 5. It was written in French** |
|  |
| Monnier, M., L. Jaunin, M. Bickle Graz, C. Borradori Tolsa, P. Hüppi, A. Sancho Rossignol, K. Barisnikov, and M. Forcada Guex. “Suivi Neurodéveloppemental à 5ans Des Extrêmes Prématurés et Détection Des Difficultés Sur Le Plan Des Fonctions Exécutives.” Archives de Pédiatrie 21, no. 9 (September 2014): 944–952. doi:10.1016/j.arcped.2014.04.032. |
|  |
| **Criterion 6. They** **were focused on executive functioning instead of executive function** |
| Cheng, Chihwen, Thomas G. Burns, and May D. Wang. “Mining Association Rules for Neurobehavioral and Motor Disorders in Children Diagnosed with Cerebral Palsy.” 2013 IEEE International Conference on Healthcare Informatics (September 2013). doi:10.1109/ichi.2013.24.  Guo, Zhiwei, Guoqiang Xing, Bin He, Huaping Chen, Jun Ou, Morgan A. McClure, Hua Liu, Yunfeng Wang, and Qiwen Mu. “Dynamic Modulation of rTMS on Functional Connectivity and Functional Network Connectivity to Children with Cerebral Palsy.” NeuroReport 27, no. 4 (March 2016): 284–288. doi:10.1097/wnr.0000000000000534.  Muriel, Vega, Antonia Ensenyat, Alberto García-Molina, Celeste Aparicio-López, and Teresa Roig-Rovira. “Déficits Cognitivos y Abordajes Terapéuticos En Parálisis Cerebral Infantil [Cognitive Deficits and Therapeutic Approaches in Children with Cerebral Palsy].” Acción Psicológica 11, no. 1 (January 19, 2015): 107. doi:10.5944/ap.11.1.13915.  Pagnozzi, Alex M., Kaikai Shen, James D. Doecke, Roslyn N. Boyd, Andrew P. Bradley, Stephen Rose, and Nicholas Dowson. “Using Ventricular Modeling to Robustly Probe Significant Deep Gray Matter Pathologies: Application to Cerebral Palsy.” Human Brain Mapping 37, no. 11 (October 6, 2016): 3795–3809. doi:10.1002/hbm.23276.  Roze, E., K. N. J. A. Van Braeckel, C. N. van der Veere, C. G. B. Maathuis, A. Martijn, and A. F. Bos. “Functional Outcome at School Age of Preterm Infants With Periventricular Hemorrhagic Infarction.” PEDIATRICS 123, no. 6 (May 26, 2009): 1493–1500. doi:10.1542/peds.2008-1919.  Scheck, Simon M., Kerstin Pannek, David A. Raffelt, Simona Fiori, Roslyn N. Boyd, and Stephen E. Rose. “Structural Connectivity of the Anterior Cingulate in Children with Unilateral Cerebral Palsy Due to White Matter Lesions.” NeuroImage: Clinical 9 (2015): 498–505. doi:10.1016/j.nicl.2015.09.014.  Sørensen, Kristian, Janne Risholm Liverød, Bjørn Lerdal, Ida E. Vestrheim, and Jon Skranes. “Executive Functions in Preschool Children with Cerebral Palsy – Assessment and Early Intervention – A Pilot Study.” Developmental Neurorehabilitation (May 19, 2014): 1–6. doi:10.3109/17518423.2014.916761.  Stadskleiv, Kristine, Reidun Jahnsen, and Stephen von Tetzchner. “Structure of Executive Functioning in Children with Cerebral Palsy: An Investigation of Anderson’s Developmental Model.” Journal of Developmental and Physical Disabilities 28, no. 5 (July 5, 2016): 665–684. doi:10.1007/s10882-016-9500-6. |
| Tervo, Raymond C., Frank Symons, Jean Stout, and Tom Novacheck. “Parental Report of Pain and Associated Limitations in Ambulatory Children With Cerebral Palsy.” Archives of Physical Medicine and Rehabilitation 87, no. 7 (July 2006): 928–934. doi:10.1016/j.apmr.2006.02.023.  Van Rooijen, M., L. Verhoeven, and B. Steenbergen. “From Numeracy to Arithmetic: Precursors of Arithmetic Performance in Children with Cerebral Palsy from 6 till 8 Years of Age.” Research in Developmental Disabilities 45–46 (October 2015): 49–57. doi:10.1016/j.ridd.2015.07.001.  Whittingham, Koa, Harriet L Bodimeade, Owen Lloyd, and Roslyn N Boyd. “Everyday Psychological Functioning in Children with Unilateral Cerebral Palsy: Does Executive Functioning Play a Role?” Developmental Medicine & Child Neurology 56, no. 6 (January 7, 2014): 572–579. doi:10.1111/dmcn.12374. |
|  |

**Appendix B**. Summary of reference screening in the twelve articles included in the systematic review.

| Reference in Nadeau, Routhier, and Tessier (2008) (N = 0) | | | | | | | | |  |  |
| --- | --- | --- | --- | --- | --- | --- | --- | --- | --- | --- |
| Reference in Bottcher, Flachs and Uldall (2009) | | | | | | | | |  |  |
| Reference | | Screening by title | | | Screening by Abstract | Screening by full text | | |  |  |
|  | |  | | |  |  | | |  |  |
| Schonfeld, Amy M., Blair Paley, Fred Frankel, and Mary J. O’Connor. “Executive Functioning Predicts Social Skills Following Prenatal Alcohol Exposure.” Child Neuropsychology 12, no. 6 (December 2006): 439–452. doi:10.1080/09297040600611338. | |  | | | The sample was not comprised of at least 50% of children and adolescents with CP | X | | |  |  |
| Colver, Allan. “Study Protocol: SPARCLE – a Multi-Centre European Study of the Relationship of Environment to Participation and Quality of Life in Children with Cerebral Palsy.” BMC Public Health 6, no. 1 (April 25, 2006). doi:10.1186/1471-2458-6-105. | |  | | | Did not evaluate an EF | X | | |  |  |
| Sigurdardottir, Solveig, Audur Eiriksdottir, Eva Gunnarsdottir, Marrit Meintema, Unnur Arnadottir, and Torstein Vik. “Cognitive Profile in Young Icelandic Children with Cerebral Palsy.” Developmental Medicine & Child Neurology 50, no. 5 (May 2008): 357–362. doi:10.1111/j.1469-8749.2008.02046.x. | |  | | | Did not evaluate an EF | X | | |  |  |
| Anderson, Peter J., Stephen J. Wood, Dorothy E. Francis, Lee Coleman, Vicki Anderson, and Avihu Boneh. “Are Neuropsychological Impairments in Children with Early-Treated Phenylketonuria (PKU) Related to White Matter Abnormalities or Elevated Phenylalanine Levels?” Developmental Neuropsychology 32, no. 2 (September 3, 2007): 645–668. doi:10.1080/87565640701375963. | |  | | | The sample was not comprised of at least 50% of children and adolescents with CP | X | | |  |  |
| White, Desirée A., and Shawn E. Christ. “Executive Control of Learning and Memory in Children with Bilateral Spastic Cerebral Palsy.” Journal of the International Neuropsychological Society 11, no. 07 (November 2005). doi:10.1017/s1355617705051064. | |  | | |  | EF is not evaluated directly (consider errors in memory tasks as a result of inhibiting) | | |  |  |
|  | |  | | |  |  | | |  |  |
| Referenced in Jenks, van Lieshout, and Moor (2009) | | | | | | | | |  |  |
|  | |  | | |  |  | | |  |  |
| Schenker, Rony, Wendy J Coster, and Shula Parush. “Neuroimpairments, Activity Performance, and Participation in Children with Cerebral Palsy Mainstreamed in Elementary Schools.” Developmental Medicine & Child Neurology 47, no. 12 (November 16, 2005): 808-814. doi:10.1017/s0012162205001714. | |  | | | Did not evaluate an EF | X | | |  |  |
| Jenks, Kathleen M., Jan de Moor, Ernest C.D.M. van Lieshout, Karel G.B. Maathuis, Inge Keus, and Jan Willem Gorter. “The Effect of Cerebral Palsy on Arithmetic Accuracy Is Mediated by Working Memory, Intelligence, Early Numeracy, and Instruction Time.” Developmental Neuropsychology 32, no. 3 (October 18, 2007): 861–879. doi:10.1080/87565640701539758. | |  | | | Did not evaluate an EF | X | | |  |  |
| Filho, Gilberto Nunes, Lígia Souza, Luiz Guilherme Nunes, Lucia Willadino Braga, and Georges Dellatolas. “Manual Skill, Hand Skill Asymmetry, and Neuropsychological Test Performance in Schoolchildren with Spastic Cerebral Palsy.” Laterality: Asymmetries of Body, Brain and Cognition 10, no. 2 (March 2005): 161–182. doi:10.1080/13576500442000012. | |  | | | Did not evaluate an EF | X | | |  |  |
| Deary, Ian J., Steve Strand, Pauline Smith, and Cres Fernandes. “Intelligence and Educational Achievement.” Intelligence 35, no. 1 (January 2007): 13–21. doi:10.1016/j.intell.2006.02.001. | |  | | | The sample was not comprised of at least 50% of children and adolescents with CP | X | | |  |  |
| Lynn, Richard, and Jaan Mikk. “National Differences in Intelligence and Educational Attainment.” Intelligence 35, no. 2 (March 2007): 115–121. doi:10.1016/j.intell.2006.06.001. | |  | | | The sample was not comprised of at least 50% of children and adolescents with CP | X | | |  |  |
| Swanson, H. Lee. “Cognitive Processes That Underlie Mathematical Precociousness in Young Children.” Journal of Experimental Child Psychology 93, no. 3 (March 2006): 239–264. doi:10.1016/j.jecp.2005.09.006. | |  | | | The sample was not comprised of at least 50% of children and adolescents with CP | X | | |  |  |
|  | |  | | |  |  | | |  |  |
| Referenced in Pirila, Meere, Rantanen, Jokilouma, and Eriksson (2011) | | | | | | | | |  |  |
|  | |  | | |  |  | | |  |  |
| Pagliano, E., E. Fedrizzi, A. Erbetta, S. Bulgheroni, A. Solari, R. Bono, E. Fazzi, E. Andreucci, and D. Riva. “Cognitive Profiles and Visuoperceptual Abilities in Preterm and Term Spastic Diplegic Children With Periventricular Leukomalacia.” Journal of Child Neurology 22, no. 3 (March 1, 2007): 282–288. doi:10.1177/0883073807300529. | |  | | | Did not evaluate an EF | X | | |  |  |
| Pueyo, Roser, Carme Junqué, Pere Vendrell, Ana Narberhaus, and Dolors Segarra. “Neuropsychologic Impairment in Bilateral Cerebral Palsy.” Pediatric Neurology 40, no. 1 (January 2009): 19–26. doi:10.1016/j.pediatrneurol.2008.08.003. | |  | | |  | The sample of the study did not meet the criterion of age (M=22) | | |  |  |
| Reilly, Dinah S., Marjorie H. Woollacott, Paul van Donkelaar, and Sandra Saavedra. “The Interaction Between Executive Attention and Postural Control in Dual-Task Conditions: Children With Cerebral Palsy.” Archives of Physical Medicine and Rehabilitation 89, no. 5 (May 2008): 834–842. doi:10.1016/j.apmr.2007.10.023. | |  | | |  |  | | |  |  |
|  | |  | | |  |  | | |  |  |
| Referenced in Caillies, Hody, and Calmus (2012) | |  | | |  | | | |  |  |
|  | |  | | |  |  | | |  |  |
| Marcovitch, Stuart, and Philip David Zelazo. “A Hierarchical Competing Systems Model of the Emergence and Early Development of Executive Function.” Developmental Science 12, no. 1 (January 2009): 1–18. doi:10.1111/j.1467-7687.2008.00754.x. | |  | | | The sample was not comprised of at least 50% of children and adolescents with CP | X | | |  |  |
|  | |  | | |  |  | | |  |  |
| Referenced in Jenks Lieshout, and Moor (2012) | | | | | | | | |  |  |
|  | |  |  | | | |  | | |  |
| Jenks, Kathleen M., Ernest C. D. M. van Lieshout, and Jan de Moor. “The Relationship Between Medical Impairments and Arithmetic Development in Children With Cerebral Palsy.” Journal of Child Neurology 24, no. 5 (May 2009): 528–535. doi:10.1177/0883073809335009. | |  | - Did not evaluate an EF | | | | X | | |  |
|  | |  |  | | | |  | | |  |
| Referenced in Bodimeade Whittingham, Lloyd, and Boyd (2013) | |  |  | | | |  | | |  |
|  | |  |  | | | |  | | |  |
| Enkelaar, Lotte, Marjolijn Ketelaar, and Jan Willem Gorter. “Association Between Motor and Mental Functioning in Toddlers with Cerebral Palsy.” Developmental Neurorehabilitation 11, no. 4 (January 2008): 276–282. doi:10.1080/17518420802581164. |  | | | Did not evaluate an EF | | | | X | | |
| Long, B., M. M. Spencer-Smith, R. Jacobs, M. Mackay, R. Leventer, C. Barnes, and V. Anderson. “Executive Function Following Child Stroke: The Impact of Lesion Location.” Journal of Child Neurology 26, no. 3 (November 29, 2010): 279–287. doi:10.1177/0883073810380049. | |  | The sample was not comprised of at least 50% of children and adolescents with CP | | | | X | | |  |
| Dennis, Maureen, David J. Francis, Paul T. Cirino, Russell Schachar, Marcia A. Barnes, and Jack M. Fletcher. “Why IQ Is Not a Covariate in Cognitive Studies of Neurodevelopmental Disorders.” Journal of the International Neuropsychological Society 15, no. 03 (April 30, 2009): 331-343. doi:10.1017/s1355617709090481. | |  | The sample was not comprised of at least 50% of children and adolescents with CP | | | | X | | |  |
| Anderson, V., Spencer-Smith, M., Coleman, L., Anderson, P., Williams, J., Greenham, M., Leventer, R.J., Jacobs, R. “Children's executive functions: are they poorer after very early brain insult”. Neuropsychologia 48(7), (jun, 2010): 2041-2050. doi:10.1016/j.neuropsychologia.2010.03.025. | |  | The sample was not comprised of at least 50% of children and adolescents with CP | | | | X | | |  |
|  | |  |  | | | |  | | |  |
| Referenced in Dourado, Andrade, Ramos-Jorge, Moreira, and Oliveira-Ferreira (2013) | |  |  | | | |  | | |  |
|  | |  |  | | | |  | | |  |
| Trick, Lana M., Rachna Mutreja, and Kelly Hunt. “Spatial and Visuospatial Working Memory Tests Predict Performance in Classic Multiple-Object Tracking in Young Adults, but Nonspatial Measures of the Executive Do Not.” Attention, Perception, & Psychophysics 74, no. 2 (November 11, 2011): 300–311. doi:10.3758/s13414-011-0235-2. | |  | The sample was not comprised of at least 50% of children and adolescents with CP | | | | X | | |  |
| Vaquero, Encarna, Carlos M Gómez, Eliana A Quintero, Javier J González-Rosa, and Javier Márquez. “Differential Prefrontal-Like Deficit in Children after Cerebellar Astrocytoma and Medulloblastoma Tumor.” Behavioral and Brain Functions 4, no. 1 (2008): 1-16. doi:10.1186/1744-9081-4-18. | |  | The sample was not comprised of at least 50% of children and adolescents with CP | | | | X | | |  |
|  | |  |  | | | |  | | |  |
| Referenced in Gofer-Levi, Silberg, Brezner, and Vakil (2014) | |  |  | | | |  | | |  |
|  | |  |  | | | |  | | |  |
| Dahlgren Sandberg, Annika. “Reading and Spelling Abilities in Children with Severe Speech Impairments and Cerebral Palsy at 6, 9, and 12 Years of Age in Relation to Cognitive Development: a Longitudinal Study.” Developmental Medicine & Child Neurology 48, no. 08 (July 12, 2006): 629-634. doi:10.1017/s0012162206001344. | |  | Did not evaluate an EF | | | | X | | |  |
| Gofer-Levi, Moran, Tamar Silberg, Amichai Brezner, and Eli Vakil. “Deficit in Implicit Motor Sequence Learning Among Children and Adolescents with Spastic Cerebral Palsy.” Research in Developmental Disabilities 34, no. 11 (November 2013): 3672–3678. doi:10.1016/j.ridd.2013.07.029. | |  | Did not evaluate an EF | | | | X | | |  |
| Huizinga, Mariëtte, Conor V. Dolan, and Maurits W. van der Molen. “Age-Related Change in Executive Function: Developmental Trends and a Latent Variable Analysis.” Neuropsychologia 44, no. 11 (January 2006): 2017–2036. doi:10.1016/j.neuropsychologia.2006.01.010. | |  | The sample was not comprised of at least 50% of children and adolescents with CP | | | | X | | |  |
|  | |  |  | | | |  | | |  |
| Referenced in Li, Wang, Wu, Hong, Zhao, Freng Xu, Wang, Min., Ndasauka, and Zhang, (2014) | | | | | | | | |  |  |
|  | |  |  | | | |  | | |  |
| Brydges, Christopher R., Corinne L. Reid, Allison M. Fox, and Mike Anderson. “A Unitary Executive Function Predicts Intelligence in Children.” Intelligence 40, no. 5 (September 2012): 458–469. doi:10.1016/j.intell.2012.05.006. | |  | The sample was not comprised of at least 50% of children and adolescents with CP | | | | X | | |  |
| Bull, Rebecca, Louise H. Phillips, and Claire A. Conway. “The Role of Control Functions in Mentalizing: Dual-Task Studies of Theory of Mind and Executive Function.” Cognition 107, no. 2 (May 2008): 663–672. doi:10.1016/j.cognition.2007.07.015. | |  | The sample was not comprised of at least 50% of children and adolescents with CP | | | | X | | |  |
| Carroll, Daniel J., Kevin J. Riggs, Ian A. Apperly, Kate Graham, and Ceara Geoghegan. “How Do Alternative Ways of Responding Influence 3- and 4-Year-Olds’ Performance on Tests of Executive Function and Theory of Mind?” Journal of Experimental Child Psychology 112, no. 3 (July 2012): 312–325. doi:10.1016/j.jecp.2012.03.001. | |  | The sample was not comprised of at least 50% of children and adolescents with CP | | | | X | | |  |
| Müller U, Liebermann-Finestone DP, Carpendale JI, Hammond SI, Bibok MB. “The effects of parental scaffolding on preschoolers' executive function”. Developmental Psychology vol. 48 (Jan 2012):271-281. doi: 10.1037/a0025519. | |  | The sample was not comprised of at least 50% of children and adolescents with CP | | | | X | | |  |
| Müller, Ulrich, Dana P. Liebermann-Finestone, Jeremy I.M. Carpendale, Stuart I. Hammond, and Maximilian B. Bibok. “Knowing Minds, Controlling Actions: The Developmental Relations Between Theory of Mind and Executive Function from 2 to 4years of Age.” Journal of Experimental Child Psychology 111, no. 2 (February 2012): 331–348. doi:10.1016/j.jecp.2011.08.014. | |  | The sample was not comprised of at least 50% of children and adolescents with CP | | | | X | | |  |
| St Clair-Thompson, Helen L., and Susan E. Gathercole. “Executive Functions and Achievements in School: Shifting, Updating, Inhibition, and Working Memory.” The Quarterly Journal of Experimental Psychology 59, no. 4 (April 2006): 745–759. doi:10.1080/17470210500162854. | |  | The sample was not comprised of at least 50% of children and adolescents with CP | | | | X | | |  |
|  | |  |  | | | |  | | |  |
| Referenced in Stadskleiv Tetzchner, Batorowicz, Balkom, and Dahlgren-Sandberg (2014) | | | | | | | | |  |  |
|  | |  |  | | | |  | | |  |
| Benson, Jeannette E., Mark A. Sabbagh, Stephanie M. Carlson, and Philip David Zelazo. “Individual Differences in Executive Functioning Predict Preschoolers’ Improvement from Theory-of-Mind Training.” Developmental Psychology 49, no. 9 (2013): 1615–1627. doi:10.1037/a0031056. | |  | The sample was not comprised of at least 50% of children and adolescents with CP | | | | X | | |  |
| Bottcher, Louise. “Children with Spastic Cerebral Palsy, Their Cognitive Functioning, and Social Participation: A Review.” Child Neuropsychology 16, no. 3 (April 21, 2010): 209–228. doi:10.1080/09297040903559630 | |  | Study were not research studies (review) | | | | X | | |  |
| Carlson, Stephanie M. “Developmentally Sensitive Measures of Executive Function in Preschool Children.” Developmental Neuropsychology 28, no. 2 (October 2005): 595–616. doi:10.1207/s15326942dn2802_3. | |  | Study were not research studies (review) | | | | X | | |  |
| Doebel, S., and Zelazo, P.D. “Bottom-up and top-down dynamics in young children's executive function: Labels aid 3-year-olds’ performance on the Dimensional Change Card Sort”. Cognitive development. Vol 28 nº 3 (jul-sep 2013): 222-232. doi: 10.1016/j.cogdev.2012.12.001 | |  | The sample was not comprised of at least 50% of children and adolescents with CP | | | | X | | |  |
| Engelhardt, Paul E., Joel T. Nigg, and Fernanda Ferreira. “Is the Fluency of Language Outputs Related to Individual Differences in Intelligence and Executive Function?” Acta Psychologica 144, no. 2 (October 2013): 424–432. doi:10.1016/j.actpsy.2013.08.002. | |  | The sample was not comprised of at least 50% of children and adolescents with CP | | | | X | | |  |
| Fatzer, Simone Tabea, and Claudia Maria Roebers. “Language and Executive Functions: The Effect of Articulatory Suppression on Executive Functioning in Children.” Journal of Cognition and Development 13, no. 4 (November 2012): 454–472. doi:10.1080/15248372.2011.608322. | |  | The sample was not comprised of at least 50% of children and adolescents with CP | | | | X | | |  |
| Foy, Judith G., and Virginia A. Mann. “Executive Function and Early Reading Skills.” Reading and Writing 26, no. 3 (April 25, 2012): 453–472. doi:10.1007/s11145-012-9376-5. | |  | The sample was not comprised of at least 50% of children and adolescents with CP | | | | X | | |  |
| Ganesalingam, Kalaichelvi, Keith Owen Yeates, H. Gerry Taylor, Nicolay Chertkoff Walz, Terry Stancin, and Shari Wade. “Executive Functions and Social Competence in Young Children 6 Months Following Traumatic Brain Injury.” Neuropsychology 25, no. 4 (2011): 466–476. doi:10.1037/a0022768. | |  | The sample was not comprised of at least 50% of children and adolescents with CP | | | | X | | |  |
| Usai, M. Carmen, Paola Viterbori, Laura Traverso, and Valentina De Franchis. “Latent Structure of Executive Function in Five- and Six-Year-Old Children: A Longitudinal Study.” European Journal of Developmental Psychology 11, no. 4 (October 4, 2013): 447–462. doi:10.1080/17405629.2013.840578. | |  | The sample was not comprised of at least 50% of children and adolescents with CP | | | | X | | |  |
| Wiebe, S.A., Espy, K.A., Charak, D. “Using confirmatory factor analysis to understand executive control in preschool children: I. Latent structure”. Developmental Psychology vol 44 nº2 (Mar 2008): 575-587. doi: 10.1037/0012-1649.44.2.575. | |  | The sample was not comprised of at least 50% of children and adolescents with CP | | | | X | | |  |
| Willoughby, Michael, and Clancy Blair. “Test-Retest Reliability of a New Executive Function Battery for Use in Early Childhood.” Child Neuropsychology 17, no. 6 (November 2011): 564–579. doi:10.1080/09297049.2011.554390. | |  | The sample was not comprised of at least 50% of children and adolescents with CP | | | | X | | |  |
|  | |  |  | | | |  | | |  |
| Referenced in Piovesana, Stephanie, Whittingham, Ware, and Boyd (2015) | |  |  | | | |  | | |  |
| Anderson, V., M. Spencer-Smith, and A. Wood. “Do Children Really Recover Better? Neurobehavioural Plasticity after Early Brain Insult.” Brain 134, no. 8 (July 22, 2011): 2197–2221. doi:10.1093/brain/awr103. | |  | Study were not research studies (review) | | | | X | | |  |
| Boyd, Roslyn N, Louise E Mitchell, Sarah T James, Jenny Ziviani, Leanne Sakzewski, Anthony Smith, Stephen Rose, et al. “Move It to Improve It (Mitii): Study Protocol of a Randomised Controlled Trial of a Novel Web-Based Multimodal Training Program for Children and Adolescents with Cerebral Palsy.” BMJ Open 3, no. 4 (2013): e002853. doi:10.1136/bmjopen-2013-002853. | |  | ✓ | | | | Did not evaluate an EF | | |  |
| Burnett, Alice Claudia, Shannon Elizabeth Scratch, and Peter John Anderson. “Executive Function Outcome in Preterm Adolescents.” Early Human Development 89, no. 4 (April 2013): 215–220. doi:10.1016/j.earlhumdev.2013.01.013. | |  | The sample was not comprised of at least 50% of children and adolescents with CP | | | | X | | |  |
| Edgin, Jamie O., Terrie E. Inder, Peter J. Anderson, Kelly M. Hood, Caron A.C. Clark, and Lianne J. Woodward. “Executive Functioning in Preschool Children Born Very Preterm: Relationship with Early White Matter Pathology.” Journal of the International Neuropsychological Society 14, no. 01 (December 14, 2007):90-101. doi:10.1017/s1355617708080053. | |  | The sample was not comprised of at least 50% of children and adolescents with CP | | | | X | | |  |
|  | |  |  | | | |  | | |  |
| Referenced in Ballester-Plane et al. | |  |  | | | |  | | |  |
| Baglio, Francesca, Monia Cabinio, Cristian Ricci, Gisella Baglio, Susanna Lipari, Ludovica Griffanti, Maria G. Preti, et al. “Abnormal Development of Sensory-Motor, Visual Temporal and Parahippocampal Cortex in Children with Learning Disabilities and Borderline Intellectual Functioning.” Frontiers in Human Neuroscience 8 (October 15, 2014). doi:10.3389/fnhum.2014.00806. | |  | Did not evaluated an EF | | | | X | | |  |
| Himmelmann, Kate, and Paul Uvebrant. “Function and Neuroimaging in Cerebral Palsy: a Population-Based Study.” Developmental Medicine & Child Neurology 53, no. 6 (May 16, 2011): 516–521. doi:10.1111/j.1469-8749.2011.03932.x | |  | Did not evaluated an EF | | | | X | | |  |
| Mullen, Katherine M., Betty R. Vohr, Karol H. Katz, Karen C. Schneider, Cheryl Lacadie, Michelle Hampson, Robert W. Makuch, Allan L. Reiss, R. Todd Constable, and Laura R. Ment. “Preterm Birth Results in Alterations in Neural Connectivity at Age 16 Years.” NeuroImage 54, no. 4 (February 2011): 2563–2570. doi:10.1016/j.neuroimage.2010.11.019. | |  | Did not evaluated an EF | | | | X | | |  |
| Peeters, M., L. Verhoeven, H. van Balkom, and J. de Moor. “Foundations of Phonological Awareness in Pre-School Children with Cerebral Palsy: The Impact of Intellectual Disability.” Journal of Intellectual Disability Research 0, no. 0 (August 8, 2007): 070808045409005–??? doi:10.1111/j.1365-2788.2007.00986.x. | |  | Did not evaluated an EF | | | | X | | |  |
| Rai, Yogita, Saurabh Chaturvedi, Vimal Kumar Paliwal, Puneet Goyal, Ankita Chourasia, Ram Kishore Singh Rathore, Abhishek Yadav, et al. “DTI Correlates of Cognition in Term Children with Spastic Diplegic Cerebral Palsy.” European Journal of Paediatric Neurology 17, no. 3 (May 2013): 294–301. doi:10.1016/j.ejpn.2012.11.005. | |  |  | | | | Did not evaluated an EF | | |  |
| Robinson, Marnie N, Lyndal J Peake, Michael R Ditchfield, Susan M Reid, Anna Lanigan, and Dinah S Reddihough. “Magnetic Resonance Imaging Findings in a Population-Based Cohort of Children with Cerebral Palsy.” Developmental Medicine & Child Neurology 51, no. 1 (January 2009): 39–45. doi:10.1111/j.1469-8749.2008.03127.x. | |  |  | | | | Did not evaluated an EF | | |  |
| Wells, Carolyn T., E. Mark Mahone, Melissa A. Matson, Wendy R. Kates, Trisha Hay, and Alena Horska. “Relationship of Temporal Lobe Volumes to Neuropsychological Test Performance in Healthy Children.” Brain and Cognition 68, no. 2 (November 2008): 171–179. doi:10.1016/j.bandc.2008.04.004. | |  | The sample was not comprised of at least 50% of children and adolescents with CP | | | | X | | |  |
| Referenced in Laporta-Hoyos et al. | |  |  | | | |  | | |  |
| Mulder, Hanna, Huub Hoofs, Josje Verhagen, Ineke van der Veen, and Paul P. M. Leseman. “Psychometric Properties and Convergent and Predictive Validity of an Executive Function Test Battery for Two-Year-Olds.” Frontiers in Psychology 5 (July 22, 2014). doi:10.3389/fpsyg.2014.00733. | |  | The sample was not comprised of at least 50% of children and adolescents with CP | | | | X | | |  |
| Referenced in Piovesana et al. | |  |  | | | |  | | |  |
| “Working Memory Training Improves Cognitive Function in VLBW Preschoolers.” PEDIATRICS 131, no. 3 (February 11, 2013): X22–X22. doi:10.1542/peds.2012-1965d. | |  |  | | | | Did not evaluated an EF | | |  |
| Bleyenheuft, Yannick, Laurence Dricot, Nathalie Gilis, Hsing-Ching Kuo, Cécile Grandin, Corinne Bleyenheuft, Andrew M. Gordon, and Kathleen M. Friel. “Capturing Neuroplastic Changes after Bimanual Intensive Rehabilitation in Children with Unilateral Spastic Cerebral Palsy: A Combined DTI, TMS and fMRI Pilot Study.” Research in Developmental Disabilities 43–44 (August 2015): 136–149. doi:10.1016/j.ridd.2015.06.014. | |  | Did not evaluated an EF | | | | X | | |  |

| **Appendix C.** Summary of the studies included in the systematic review | | | |  |  |  |  |  |
| --- | --- | --- | --- | --- | --- | --- | --- | --- |
| **References** | **# individuals CP**  **(% of total sample with CP, EG/CG*);**  **Mean age/**  **Age Range/ Median (SD)** | **Characteristics of the sample with CP (Motor classification; gender)** | **Objective (design)** | | **EFs Assessed** | **Instrument / Task** | **Other areas/domains assessed** | **Results** |
|  |  |  |  | |  |  |  |  |
| Nadeau, Routhier, & Tessier (2008) [45] | N = 52 (100 EG^+^);  11.4 (0.98)  N = 50 (CG^++^);  11 (0.95) | Type/Subtype: Right Hemiplegia (N = 37; 71%) and Diplegia (N = 15; 29%);  Gender: Hemiplegia (26 boys); Diplegia (5 boys)  GMFCS (Level I to V): I;  Global IQ (WISC-III): M = 93.7; SD = 16.1;  Mainstream School Students. | To compare children with CP and children without impairment in the performance profile on the Wisconsin Card Sorting Test.  (Longitudinal study) | | Cognitive Flexibility | Wisconsin Card Sorting Test (WCST) | NA | The results indicate that children with CP: i) make more non-perseverative errors, ii) completed fewer categories, iii) required more trials to complete the first category, and iv) gave fewer conceptual responses than children without impairment. The difficulty may be due to a slow rate of information processing and/or cognitive deficits.  Total number of errors (TE)  Non-perseverative errors (NpE)  Number of categories completed (NCC)  Trials to complete first category (TFC)  Conceptual level responses (CLR)  Diplegia: TE*; NpE*, NCC**, CLR*  Hemiplegia: NpE* |
|  |  |  |  | |  |  |  |  |
| Reilly, Woollacott, Donkelaar, & Saavedra (2008) [46]  (reference screening) | N = 8 (100 EG^+^);  10-14  N = 11 (CG^++^);  4-12 | Type/Subtype: Spastic CP (N = 4; 2 boys) and Ataxic CP (N = 4; 2 boys);  GMFCS (Level I to V): I (N = 4), II (N = 1), III (N = 3). | To study the interference between a secondary and postural task in children with CP.  (Exploratory Study) | | Attention | Dual-task (Postural control task with an attentional challenging cognitive task– interference task);  Visual working memory task;  COP Movement in Single and dual task conditions | NA | Similarly to typically developing young children, children with CP were unstable and had less executive attention capacity compared with older children. Moreover, children with CP, like the typically developing young children, experienced dual-task interference in postural control in both stance positions. Specifically, children with ataxic CP also experienced decreased cognitive task performance in narrow stance.  Dual-task, wide stance  CP like the young typically developing children experience dual-task interference in the postural control (anteroposterior (AP) range**, range and the root mean square (RMS) AP velocity***, and RMS mediolateral (ML) velocity*)  Dual-task, narrow stance  CP like the young typically developing children experience dual-task interference in the postural control (ML range**, RMS ML velocity*, RMS AP velocity***) |
|  |  |  |  | |  |  |  |  |
| Bottcher, Flachs, & Uldall (2009) [42] | N = 33 (100 EG^+^); 11.3 (1.2)  Norm test population | Type/Subtype: Unilateral Spastic (N = 15) and Bilateral Spastic (N = 18).  Gender: males (N = 19).  GMFCS (Level I to V): I (N = 22), II (N = 3), III (N = 6), IV (N = 2);  Epilepsy (N = 3)  Mainstream School (N = 26), Mainstream School with Assistance (N = 3); Special Class School (N = 5). | To investigate if children with CP have impairments in attention or EFs.  (Cross-sectional Study) | | Attention  Inhibition  Inhibition  Shifting  Emotional Control  Initiating  Working Memory  Planning/ Organizing  Organization of Materials  Monitoring | Test of Everyday Attention for Children (TEA-Ch): Sky Search, Score!, and Sky Search Dual Task  Contingency Naming Test (CNT)  BRIEF | NA | Verbal cognitive functioning was within the normative results, whereas sustained and divided attention was found to be impaired in children with CP. The EF shows greater impairment than attention function, particularly in inhibition and shifting functions. No significant differences were found between types of CP (unilateral and bilateral). Performance in all timed tasks was slower than the test norm.  Comparison between unilateral CP, bilateral CP, all spastic CP and norm population in verbal comprehension, attention measures and executive function results.  Verbal comprehension and attention (CP vs norm)  No statistically significant difference was found between unilateral and bilateral CP.  VCI (WISC-III), *p* = 0.10.  TEA-Ch (Sky Search C***, Sky Search G***, Socre!***, Sky Search Dual Task***) .  Executive Function (Cp vs norm)  CNT subtest: 1 (time and efficiency***), 2 (time and efficiency***), 3 (time and efficiency***), 4 (time***).  BRIEF (Shift***, Emotional control***, Behavioral Index***, Initiate***, Working Memory***, Plan/organize***, Monitor***, Metacognition index***, General executive function***). |
|  |  |  |  | |  |  |  |  |
| Jenks, van Lieshout, & Moor (2009) [47] | N = 41 (100 CP special^++++^);  7.0 (0.7)  N = 16 (100 CP mainstream^++++^);  7.0 (0.4)  N = 16 (Control^+++^); 6.9 (0.4) | CP Special School (N = 41; males = 68.3%)  Type/Subtype: Right Unilateral Spastic (12.2%), Left Unilateral Spastic CP (12.2%), Bilateral Spastic CP (70.7%), Ataxic CP (4.9%).  GMFCS (Level I to V): I (26.8%), II (24.4%), III (22%), IV (24.3%), V (2.4%).  Visual Impairments (36.5%)  Hearing Impairments (4.9%)  Epilepsy (24.4%).  CP Mainstream School (N = 16; males = 56.3%)  Type/Subtype: Right Unilateral Spastic (43.8%), Left Unilateral Spastic CP (31.3%), Bilateral Spastic CP (18.8%), Ataxic CP (6.3%).  GMFCS (Level I to V): I (62.5%), II (12.5%), III (25%).  Visual Impairments (25%)  Epilepsy (12.5%). | First goal: to study developmental arithmetic ability in children with CP.  Second goal: to study whether arithmetic ability could be predicted by the severity of motor impairment, localization of CP, visual and hearing impairment, epilepsy, verbal and nonverbal intelligence, and the differences between two CP school groups (mainstream and special)  (Cross-sectional Study) | | Inhibition and Shifting  Updating  Visuospatial sketchpad  Phonological Loop | Shifting-naming and Inhibition-naming  (van der Sluis, de Jong, and van der Leij)  Backwards Digits (Working Memory  Test Battery for Children)  Knox blocks (subtest of the Snijders-Oomen Non Verbal Intelligence Test)  Both Digit Recall and Word Recall (Working Memory Test Battery for Children) | NA | Children with CP had lower accuracy and consistently slower (verbal) response times, which raises concerns for their future arithmetic development. Differences in arithmetic performance between children with CP in special, or mainstream, education were not related to location of CP or to gross motor impairment. Rather, lower accuracy and slower verbal responses were related to differences in nonverbal intelligence and the presence of epilepsy. Left-hand impairment was related to slower verbal responses but not to lower accuracy.  *Development of Arithmetic Accuracy*  CP special less accuracy than CP mainstream and Control***.  No statistically significant difference was found between CP mainstream and Control  *Development of Arithmetic Speed (Response Time)*  CP special have a slower performance on both addition and subtraction than CP mainstream and Control**.  No statistically significant difference was found between CP mainstream and Control. |
|  |  |  |  | |  |  |  |  |
| Pirila, Meere, Rantanen, Jokilouma, & Eriksson (2011) [48] | N = 17 (100 EG^+^)  13.5 (2.8)  /8-17 | Type/Subtype: Bilateral Spastic CP (N = 9), Unilateral Spastic CP (N = 8);  Gender: NR.  GMFCS (Level I to V): I (N = 12), II (N = 1), III (N = 4).  MACS (Level I to V): I (N = 11), II (N = 5), III (N = 1). | To investigate whether spastic CP is associated with impaired EFs in clinical range.  (Cross-sectional Study) | | Attention | Conners Continues Performance Test | NA | Patients with CP, bilateral lesions, and preterm birth are associated with executive function problems in the clinical range.  Negative correlation between birth weight and executive function classification**  40% of the variation of severity of the executive function problems was explained by birth weight* |
|  |  |  |  | |  |  |  |  |
| Caillies, Hody, & Calmus (2012) [49] | N = 10 (100 EG^+^);  7-11  N = 10 (CG^++^);  7-11 | Type/Subtype: Spastic Diplegia (N = 5), Right Spastic Hemiplegia (N = 4), Ataxic CP (N = 1).  Gender: male (N=6).  Global IQ (WISC-IV) (N = 10) > 85. | To characterize the pragmatic abilities of French children with CP, appealing the comprehension of irony and other people’s mental states.  (Cross-sectional Study) | | Working Memory  Inhibitory control | Digit Span Forward and Backward  Letter-Number Sequencing (WISC-IV)  Stroop Test  Knock-Tap Test (A Developmental NEuroPSYchological Assessment - NEPSY) | Theory of Mind (ToM)  Irony comprehension  Language comprehension | Children with CP show difficulties in pragmatic skills. The groups differed significantly on second-order theory of mind, irony comprehension, and working memory.  Group performances in the Letter–Number Sequencing task (WISC-IV)*  Correlations:  Digit Span/ Speaker’s belief (Other-directed irony)*  Letter–Number Sequencing / Speaker’s belief (Other-directed irony)* |
|  |  |  |  | |  |  |  |  |
| Jenks Lieshout, & Moor (2012) [50] | N = 41 (100 EG^+^ – CP special^++++^);  8.93 (0.79)  N = 16 (100 EG^+^ – CP mainstream^++++^); 8.88 (0.34)  N = 16 (CG^++^ – Control^++++^); 8.94 (0.44) | Gender: matched in each group in all groups.  CP Special School (N = 41).  Type/Subtype: Right Unilateral Spastic (12.2%), Left Unilateral Spastic CP (12.2%), Bilateral Spastic CP (70.7%), Ataxic CP (4.9%).  Verbal IQ (Peabody Picture Vocabulary Test-Revised): M = 89.9, SD = 14.5  Non-verbal IQ (Raven’s Colored Progressive Matrixes): M = 85.4, SD = 14.4  CP Mainstream School (N = 16)  Type/Subtype: Right Unilateral Spastic (43.8%), Left Unilateral Spastic CP (31.3%), Bilateral Spastic CP (18.8%), Ataxic CP (6.3%).  Verbal IQ (Peabody Picture Vocabulary Test-Revised): M = 93, SD = 16.5  Non-verbal IQ (Raven’s Colored Progressive Matrixes): M = 98.3, SD = 16.7 | To investigate the word-problem solving ability through math achievement and reading skills.  (Cross-sectional Study) | | Phonological Loop of Working Memory  Visuospatial sketchpad  Updating  Inhibition and Shifting | Digit Recall (subtest of Working Memory Test Battery for Children)  Knox Blocks (subtest of the Snijders-Oomen Non Verbal Intelligence Test)  Backwards Digits (subtest of Working Memory Test Battery for Children)  Shifting-naming and Inhibition-naming  (van der Sluis, de Jong, and van der Leij) | Arithmetic fact fluency  Three-Minute Reading test  Mathematical achievement | Children with CP in special schools have exhibited more difficulties than their peers on word-problem solving and reading. Children with CP in mainstream schools have shown a trend to a worse performance when compared to typically developing children.  Effect of group on third grade mathematics  achievement while controlling for verbal and non-verbal IQ*  The CP special scored lower than both the CP mainstream group and  Control*  No statistically significant difference was found between CP mainstream and Control.  The ANCOVA on third grade performance on the Three-Minute Reading test showed a small to medium-sized effect of group.  The expected direction (CP special < CP mainstream < Control).  EF predictors of math achievement:  Working Memory and EF   - Phonological loop* - Visuospatial sketchpad** - Updating** - Shifting control**   EF predictors of reading:  Working Memory and EF   - Phonological loop** - Visuospatial sketchpad** - Updating** - Shifting RT (-)** |
|  |  |  |  | |  |  |  |  |
| Bodimeade Whittingham, Lloyd, & Boyd (2013) [23] | N = 24 (100 EG1^+^);  11.08 (2.38)  N = 22 (100 EG2^+^); 11.09 (2.54)  N = 20 (CG^++^);  10.8 (2.29) | Unilateral CP (N = 46), Right side (N = 22).  Gender: males (N = 25).  GMFCS (Level I to V): I (N = 14), II (N = 8).  MACS (Level I to V): I (N = 1), II (N = 21).  Global IQ (WISC-IV): M = 84.94, SD = 14.65.  Seizure Disorder (controlled) (N = 3);  Learning Disorder (N = 6);  Hearing Impairment (N =1);  Vision Impairment (N = 6);  ADHD (N = 3);  Autism Spectrum Disorder (N =2);  Anxiety Disorder (N =1)  Perthes disease (N =1).  Left Unilateral CP (N =24)  GMFCS (Level I to V): I (N = 21), II (N = 3).  MACS (Level I to V): I (N = 5), II (N = 19).  Global IQ (WISC-IV): M = 86.75, SD = 17.96.  Seizure Disorder (controlled) (N = 4);  Learning Disorder (N = 7);  Hearing Impairment (N =3);  Vision Impairment (N = 9);  ADHD (N = 3);  Autism Spectrum Disorder (N =3). | To compare EFs in children with left (EG1) and right (EG2) sided unilateral CP with typically developing children.  (Cross-sectional Study) | | Cognitive Flexibility  Goal Setting  Information Processing  Attentional Control | Digit Span Backward (WISC-IV)  Trail Making Test (D-KEFS)  Verbal Fluency Test (D-KEFS)  Color-Word Interference Test (D-KEFS)  Verbal Fluency Test (D-KEFS)  Rey-Osterrieth Complex Figure Test  Tower Test (D-KEFS)  Symbol Search (WISC-IV)  Cancellation (WISC-IV)  Code Transmission Test (TEA-Ch)  Trail Making Test, Verbal Fluency and Color-Word Interference Test (D-KEFS) | NA | Children with CP, compared with typically developing children, showed worse performance on all measures of executive function. There were no significant differences between children with left and right unilateral CP except in the case of inhibition/switching total errors, with children with left unilateral CP making fewer errors than children with right unilateral CP.  Poor performance on EF measures in CP group than Control group***  Left and right CP show differences in inhibition/switching total errors* |
|  |  |  |  | |  |  |  |  |
| Dourado, Andrade, Ramos-Jorge, Moreira, & Oliveira-Ferreira (2013) [51] | N = 76 (100 EG^+^); 8.9 (3.56)  N = 89 (CG^++^); 8.9 (3.56) | Type/Subtype: Spastic CP (N = 76);  Gender: NR.  GMFCS (Level I to V): I (63.2%), II (10.3%), III (14.7%), IV (8.9%). | To evaluate the presence of an association between attention/EFs and the development of dental caries in children with CP.  (Cross-sectional Study) | | Working Memory | Digit Span Test Backward (WISC-IV)  Corsi Blocks Task Backward  Rey-Osterrieth Complex Figure Test | NA | Children with CP, compared with the control group, showed a significantly lower performance on intelligence, attentional function, and EFs tests. After controlling for intellectual function, clinical diagnosis, and motor impairment, deficits in executive and attentional functions increased the odds of developing dental caries in children with CP.  CP group lower performance on EF tests than Control group*** |
|  |  |  |  | |  |  |  |  |
| Gofer-Levi, Silberg, Brezner, & Vakil (2014) [52] | N = 24 (100 EG^+^);  9-20/*M*=13 (3.51)  N = 24 (CG^++^);  9-18/*M*=12.92 (3.52) | Type/Subtype: Bilateral Spastic CP.  Gender: males (N = 12).  GMFCS (Level I to V): I (N = 5), II (N = 5), III (N = 10), IV (N = 4).  Intelligence Ability (Raven’s Colored Progressive Matrixes), IQ *Z* scores (M = -1.75, SD = 1.56) | To examine the ability of children and adolescents with CP to develop a specific cognitive competence as an indicator of their ability for procedural learning.  (Case Control Study) | | Cognitive flexibility | Children Category Test (CCT)/ Booklet Category Test (BCT) | Probabilistic learning | In learning of Probabilistic (cognitive competence) was observed an improvement for the two groups, with no relation to IQ level. There was a difference in learning curves of children with CP on a cognitive procedural learning task compared with typical developing peers. These results do not appear to be age sensitive.  EF was not related to the level of improvement on the PCL task in the TD group nor in the CP group. |
|  |  |  |  | |  |  |  |  |
| Li, Wang, Wu, Hong, Zhao, Freng Xu, Wang, Min., Ndasauka, & Zhang, (2014) [53] | N = 42 (100 EG^+^); 10.40 (1.45)  N = 42 (CG^++^) matched for age with EG | Type/Subtype: Hemiplegia Spastic CP (N = 18), Diplegia Spastic CP (N = 13), Atethoid CP (N = 4), Ataxic CP (N = 3), Mixed CP (N = 4).  Gender: males (N = 25).  Global IQ (Chinese WISC): M = 91.57, SD = 8.69.  Intelligence Ability (Raven’s Colored Progressive Matrixes): (M = 17.97, SD = 6.69). | To explore the relation between deficits in the theory of mind and EFs impairments in children with CP.  (Mixed Study) | | Inhibition  Updating  Shifting | The inhibitory ability task  The information updating task  The attention shifting task | ToM | In theory of mind and EFs tasks, children with CP had a lower performance than typically developing children. There was a strong correlation in two EFs components (inhibition and updating) and false belief, and faux pas, in both groups (EG and CG). However the correlation with shifting component was only present in typically developing children.  EFs tasks:  Inhibition task - CP children had a poorer TD children**;  Updating task - CP children scored less than  TD children**;  Shifting task - CP children had a poorer performance than the TD children***.  Relationship between theory of mind, IQ and EF in the CP group:   - Inhibition/False belief (-)**; - Updating/False belief**; - Inhibition/Faux pas (-)* - Updating/Faux pas**; |
|  |  |  |  | |  |  |  |  |
| Stadskleiv, Tetzchner, Batorowicz, Balkom, & Dahlgren-Sandberg (2014) [54] | N = 29 (93 EG^+^); 11.34 (2.81)  N = 27 (CG^++^);  11.43 (2.63) | Type/Subtype: CP (N = 27).  Gender: male (40.7%)  Aided Communication (N = 29)  GMFCS (Level I to V): I (N = 1), II (N = 0), III (N = 2), IV (N = 9), V (N = 17).  MACS (Level I to V): I (N = 1), II (N = 1), III (N = 2), IV (N = 8), V (N =17).  Viking Speech Scale Level (Level I to IV): I (N = 0), II (N = 0), III (N = 1), IV (N = 28).  CFCS (Level I to V): I (N = 0), II (N = 13), III (N = 8), IV (N = 2), V (N = 0). | To investigate EFs in children with severe problems in speech and motor impairments.  (Cross-sectional Study) | | Planning  Monitoring  Impulsivity | Becoming an Aided Communicator (BAC) with two tasks: construction (BAC Construction) and description without naming (BAC Description without Naming) | NA | The results showed that aided communicators had a lower performance on planning skills and displayed more impulsivity level than the control group. The findings increased the perspective on the EFs impact and the implications for intervention with motor-impaired children developing aided communication. |
|  |  |  |  | |  |  |  |  |
| Piovesana, Stephanie, Whittingham, Ware, & Boyd (2015) [19] | N = 44 (100 EG^+^); 11.96 (2.47) | Type/Subtype: Unilateral CP (N = 44).  Gender: males (N = 21).  GMFCS (Level I to V): I (N = 22), II (N = 22).  MACS (Level I to V): I (N = 6), II (N = 37).  Global IQ (WISC-IV): M = 79.33, SD = 20.23.  Intellectual Disability (N = 7);  Learning Difficulties (N = 9);  Autism (N = 1);  ADHD (N = 5);  Vision Impairment (N = 6);  Hearing Impairment (N = 3);  Epilepsy (controlled) (N = 7). | To study the stability of EFs measures in children and adolescents with mild to moderate unilateral CP.  (Test-retest Study) | | Working Memory  Attention Control  Goal Setting  Processing Speed  Inhibition  Shifting  Emotional Control  Initiating  Working Memory  Planning/ Organizing  Organization of Materials  Monitoring | Working Memory Index (WISC-IV-SF)  Color-Word interference test (D-KEFS)  Trail Making Test (D-KEFS)  The Digit Span Backwards (WISC-IV)  The Coding and Symbol Search (WISC-IV)    BRIEF | NA | The results support the hypothesis of stability over time of EFs measures in this specific population. The results specified excellent scores to fair test–retest reliabilities for all measures except Digit Span Backwards, Inhibition, and Initiate. Reliable change scores applying 90% confidence intervals for estimating reliable change while accounting for practice effects were provided for all measures.  Excellent to fair Pearson’ s test– retest reliabilities (r = 0.91– 0.74) in most of the measures used, confirming the stability over testing periods in CP. |
| Ballester-Plane, Laporta-Hoyos, Macaya, Poo, Melendez-Plumed, Vazquez, Delgado, Zubiaurre-Elorza, Narberhaus, Toro-Tamargo, Russi, Tenorio, Segarra, & Pueyo (2016) [55] | Total sample  N = 44 (100 EG^+^);  23.05 (11.23); 8-51  Neuroimaging sample  N = 30 (100 EG^+^);  25.10 (12.05), 10-51 | Type/Subtype: Bilateral dyskinetic CP.  Gender: males (N = 26).  Epilepsy (N = 20);  GMFCS (Level I to V): I (N = 9), II (N = 6), III (N = 6), IV (N = 10), V (N = 13).  BFMF (Level I to V): I (N = 4), II (N = 8), III (N = 6), IV (N = 12), V (N = 6).  MACS (Level I to V): I (N = 2), II (N = 7), III (N = 14), IV (N = 11), V (N = 10).  CFCS (Level I to V): I (N = 12), II (N = 19), III (N = 8), IV (N = 5).  Intelligence (Mean, SD)/Range:  RCPM (84.23, 23.85)/ 20-117;  PPVT-III (67.77, 17.83)/55-119;  WNV (79.35, 25.35)/38-123  Mainstream school (N = 25);  Special School (N = 19).  Type/Subtype: Bilateral dyskinetic CP.  Gender: males (N=20).  Epilepsy (N = 11);  GMFCS (Level I to V): I (N = 9), II (N = 6), III (N = 3), IV (N = 4); V (N = 8).  BFMF (Level I to V): I (N = 4), II (N = 7), III (N = 10), IV (N = 7), V (N = 2).  MACS (Level I to V): I (N = 2), II (N = 7), III (N = 11), IV (N = 4), V (N = 6).  CFCS (Level I to V): I (N = 11), II (N = 13), III (N = 4), IV (N = 2).  Intelligence (Mean, SD)/Range:  RCPM (83.53, 20.95)/ 53-117;  PPVT-III (69.13, 19.34)/55-119;  WNV (79.17, 24.45)/38-123  Mainstream school (N = 19);  Special School (N = 11) | To compare three intelligence tests and understand which are more appropriated to use with CP population.  Moreover, to study the relationship between the measures and the performance in specific cognitive functions and brain volume data.  (Mixed Study) | | Cognitive Flexibility  Planning and problem solving ability  Risk taking behavior  MRI acquisition | Wisconsin Card Sorting Test (WCST)  Stockings of Cambridge subtest of CANTAB  Balloon Analogue Risk Task youth version (BART-Y)  Siemens Magnetom TRIO 3.0 T scanner. High-resolution three-dimensional T1-weighted images were acquired in the sagittal plane with a MPRAGE sequence (TR/TE 1900/2.46 ms;TI 900 ms; flip angle 9◦; 320 × _307 matrix and voxel size 0.7 mm × _0.7 mm × _1 mm). | Motor function impairment  Attention  Language  Visuoperception  Memory | The results suggests that all subjects included in the study, even severe cases, were able to complete the two single-task tests, RCPM and PPVT-III. EF were not associated with any measure of intellligence tested.  Significant difference in the mean scores of the three tests (χ^2^_F_ (2) = 28.86)**.  EF were not significantly associated with measures of intelligence in any model of the regression analysis. |
| Laporta-Hoyos, Ballester-Plané, Póo, Macaya, Meléndez-Plumed, Vázquez, Delgado, Zubiaurre-Elorza, Botellero, Narberhaus, Toro-Tamargo, Segarra & Pueyo (2016) [56] | General Sample  N = 50 (100 EG^+^); 25.96 (12.41)/12–62  Neuroimaging Sample  N = 36 (100 EG^+^);  27.81 (13.42)/12–62 | Type/Subtype: Tetraplegia (N = 42), hemiplegia (N = 7), monoplegia (N = 1)  Gender: males (N = 27).  Type/Subtype:  Tetraplegia (N = 30), hemiplegia (N = 5), monoplegia (N = 1).  Gender: males (N = 20). | The study aimed (1) to investigate the impact of different variables (gross and fine motor status, communication,  IQ, executive functions, anxiety and depressive  symptoms and socioeconomic status) on quality of life in adolescents and adults with CP and (2) to identify neuroanatomical  areas related to the CP QOL total score in  adolescents and adults with CP.  (Mixed Study) | | Attention  Goal setting  Cognitive flexibility  Risk taking  MRI acquisition | WISC/WAIS  Stockings of Cambridge (SOC) test  WCST  Balloon analogue risk task (BART-Y)  MRI assessment (Siemens  MAGNETOM Trio 3T scanner).  T1-weighted images were acquired in the sagittal plane with a MPRAGE sequence  (TR/TE 1900/2.46 ms; TI 900 ms; flip angle 98 ; 320 9 307  matrix and voxel size 0.7 mm 9 0.7 mm 9 1 mm). | Quality of Life  Motor status  Communication  Cognitive ability  Anxiety and depressive symptoms  Socioeconomic status | EFs are associated with a large number of CP QOL domains. Specifically, there was observed a positive correlation and at least moderately with General well-being and participation, Communication and physical health, Family health and Feelings about functioning.  Additionally, the strongest association was found between completed categories of the WCST and the domain Feelings about functioning (large effect size).  EF (Cognitive flexibility: WCST completed categories (*z*)) x General well-being and participation**  EF ( Cognitive flexibility: WCST perseverative responses (*T*)) x General well-being and participation**  EF ( Risk taking: BART-Y (*z*)) x General well-being and participation*  EF (Cognitive flexibility: WCST perseverative responses (*T*)) x Communication and physical health)**  EF ( Risk taking: BART-Y (*z*)) x Communication and physical health)**  EF (Cognitive flexibility: WCST perseverative responses (*T*)) x Family health*  EF (Attentional control: WISC/WAIS digit span (*z*)) x Feelings about functioning**  EF (Cognitive flexibility: WCST completed categories (*z*)) x Feelings about functioning***  EF (Cognitive flexibility: WCST perseverative responses (*T*)) x Feelings about functioning*  EF (Cognitive flexibility: WCST completed categories (*z*)) x CP QOL*  EF (Cognitive flexibility: WCST perseverative responses (*T*)) x CP QOL*** |
| Piovesana, Ross, Lloyd, Whittingham, Ziviani, Ware & Boyd (2016) [57] | N = 50 (100 CG^++^);  11.86 (2.45)  N = 51 (100 EG^+^);  11.63 (2.30) | Type/Subtype: Unilateral CP - Left side (N = 20).  Gender: male (N= 25).  MACS (Level I to V): I (N = 13), II (N = 35).  GMFCS (Level I to V): I (N = 25), II (N = 25).  Intellectual disability (FSIQ < 80): N = 7.  Epilepsy (N = 7);  Learning disorder (N = 9);  Hearing Impairment (N = 3);  Vision Impairment (N = 6);  ADHD (N = 6);  Autism spectrum disorder (N = 1);  Other (N = 3).  School: Primary (N = 38), Special education (N = 2), Secondary school (N = 6), Other (N = 1).  Type/Subtype: Unilateral CP - Left side (N = 28).  Gender: male (N= 26).  MACS (Level I to V): I (N = 11), II (N = 39), III (N = 1).  GMFCS (Level I to V): I (N = 20), II (N = 31).  Intellectual disability (FSIQ < 80): N = 4.  Epilepsy (N = 11);  Learning disorder (N = 14);  Hearing Impairment (N = 1);  Vision Impairment (N = 5);  ADHD (N = 4);  Autism spectrum disorder (N = 3);  Other (N = 11).  School: Primary (N = 39), Special education (N = 2), Secondary school (N = 4), Home schooled (N = 3). | To analyze the efficacy of Move-it-to-improve-it (MitiiTM), a multi-modal web-based program, in improving EF in children with unilateral cerebral palsy (UCP).  (Randomized-control Trial Study) | | Attentional Control  Cognitive Flexibility  Goal setting  Information processing  EF in everyday life | Color-Word Interference Test (D-KEFS)  Digit Span Backwards (WISC-IV);  Number-letter Switching condition of Trail Making Test (D-KEFS).  Tower Test (D-KEFS).  Coding and Symbol Search (WISC-IV).  BRIEF | Intellectual ability | The twenty weeks multi-modal MitiiTM intervention did not improve EF in children and adolescents with UCP.  There were not observed significant differences at 20 weeks in EF capacity or performance for children who were allocated to the MitiiTM group as compared to children in the waitlist control group. |

NA – Not Assessed

NR – Not Reported

^+^EG – Experimental Group

^++^CG – Control Group

^+++^CS - Case Study

^++++^CP special – children with CP in special education; CP mainstream – Children with CP in mainstream education; Control - children with no known impairment in mainstream education.

*p ≤ 0.05; **p ≤ 0.01; ***p ≤ 0.001
